# Engineering New‐to‐Nature Biological Pathways for β,γ‐Alkanediol Synthesis

**DOI:** 10.1002/advs.202519742

**Published:** 2026-02-12

**Authors:** Haofeng Chen, Haidong Yu, Dongjiang Lin, Guojun Yang, Yuchen Xie, Yang Zhang, Jifeng Yuan

**Affiliations:** ^1^ State Key Laboratory of Cellular Stress Biology School of Life Sciences Faculty of Medicine and Life Sciences Xiamen University Fujian China

**Keywords:** alkanediols, biosynthesis, carboligation, ordinary differential equation, synthetic biology

## Abstract

β,γ‐Alkanediols are value‐added chemicals that can be used as functional solvents, biofuels, and cosmetic components. Although some linear chain β,γ‐alkanediols beside butane‐2,3‐diol are naturally present, their biosynthetic pathways remain underexplored. In this study, we expand the acetohydroxyacid synthase (AHAS)‐mediated carboligation for the synthesis of linear chain β,γ‐alkanediols in *Escherichia coli*, namely, hexane‐2,3‐diol (2,3‐HDO) and pentane‐2,3‐diol (2,3‐PDO). Two pathways for the production of 2,3‐HDO (a redesigned *Clostridium*‐derived clostridial pathway and an artificial reversal β‐oxidation pathway) were developed, and the engineered *E. coli* cells with further disruption of endogenous thioesterases produced 152.2 mm (17.98 g/L) 2,3‐HDO from 60.03 g/L glucose in the fed‐batch bioreactor. We also expand the similar design for 2,3‐PDO biosynthesis, achieving 15.5 mm (1.61 g/L) in shake flasks. This work demonstrates the great versatility of the AHAS‐mediated carboligation for the synthesis of linear chain β,γ‐alkanediols, which would pave its way for the future production of other linear chain β,γ‐diols from renewable feedstocks.

## Graphical abstract

β,γ‐Alkanediols are value‐added chemicals to be used as functional solvents, biofuels, and cosmetic components. This work demonstrates a carboligation‐mediated approach for synthesis of linear chain β,γ‐alkanediols in *Escherichia coli*, namely, hexane‐2,3‐diol (2,3‐HDO) and pentane‐2,3‐diol (2,3‐PDO). The engineered *E. coli* cells produce 152.2 mm (17.98 g/L) 2,3‐HDO in the fed‐batch bioreactor and 15.5 mm (1.61 g/L) 2,3‐PDO in shake flasks.

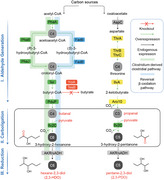

## 1 Introduction

Diols are a class of organic compounds containing two hydroxyl groups, which have a wide range of industrial applications and can be used as functional solvents [1], antifreeze [2], biofuel [3], cosmetic components [4], polymer monomers [5, 6], and drug precursors [7]. According to the different hydroxyl sites, diols can be divided into α,β‐diols, α,γ‐diols, α, ω‐diols, α,n‐diols, and β,γ‐diols [8]. The majority of diols is typically synthesized from fossil resources, which is not a sustainable method. Recently, researchers have gradually shifted their attention to sustainable and environment‐friendly fermentation production processes from renewable resources via the use of biocatalysts (including enzymes and microorganisms), to replace non‐renewable fossil resources and chemical processes [9]. To date, most of the reported microbial fermentation processes focus on the production of linear chain α,n‐alkanediols [10, 11, 12, 13, 14]. For instance, Liu et al. harnessed the highly active amino acid synthesis pathway to develop a diol biosynthetic route through a four‐step reaction for producing C3‐C5 diols [11]. Yang et al. achieved the synthesis of 1,4‐butanediol (1,4‐BDO) via the 4‐hydroxybutyryl‐CoA pathway, and achieved a titer of 20 g/L 1,4‐BDO under the optimal conditions [12]. Lee et al. explored the NADPH regeneration strategy to improve 1,3‐propanediol (1,3‐PDO) biosynthesis and achieved an 80.65 g/L 1,3‐PDO titer in fed‐batch fermentation [15]. Biobased 1,3‐PDO is currently commercialized at an industrial level by Dupont. By systematic optimization of pyruvate level and minimizing by‐products (3‐hydroxybutyric acid), Tayyab et al. engineered *Escherichia coli* to produce 1,3‐butanediol (1,3‐BDO) at a level of 70.19 g/L [16].

Beside butane‐2,3‐diol (2,3‐BDO), microbial synthesis of other β,γ‐alkanediols is generally falling behind. Although some linear chain β,γ‐alkanediols are naturally present, their biosynthetic pathways remain underexplored. Sudeep et al. isolated a strain of *Fusarium oxysporum* from the rhizome of *Curcuma amada*, and its metabolites were identified as pentane‐2,3‐diol (2,3‐PDO) by chromatographic separation and spectral analysis [17]. 2,3‐PDO has better anti‐aging properties (antioxidant, heat resistance) and has more medical application value than 1,5‐pentanediol. Feng et al. used 2,3‐PDO as an intermediate to synthesize 1,3‐pentadiene, which can be used in the production of adhesives, curing agents, and paints [18]. The longer carbon chain hexane‐2,3‐diol (2,3‐HDO), as a sex attractant and female sex pheromone of longicorn beetles, can be used as an attractant for pest control, which has great application prospects in biological control of pests [19, 20]. In addition, considering that 2,3‐PDO and 2,3‐HDO have similar chemical structures to 2,3‐BDO, they may also have great application prospects in functional solvents, biofuels, and cosmetic components [21, 22, 23].

**FIGURE 1:**
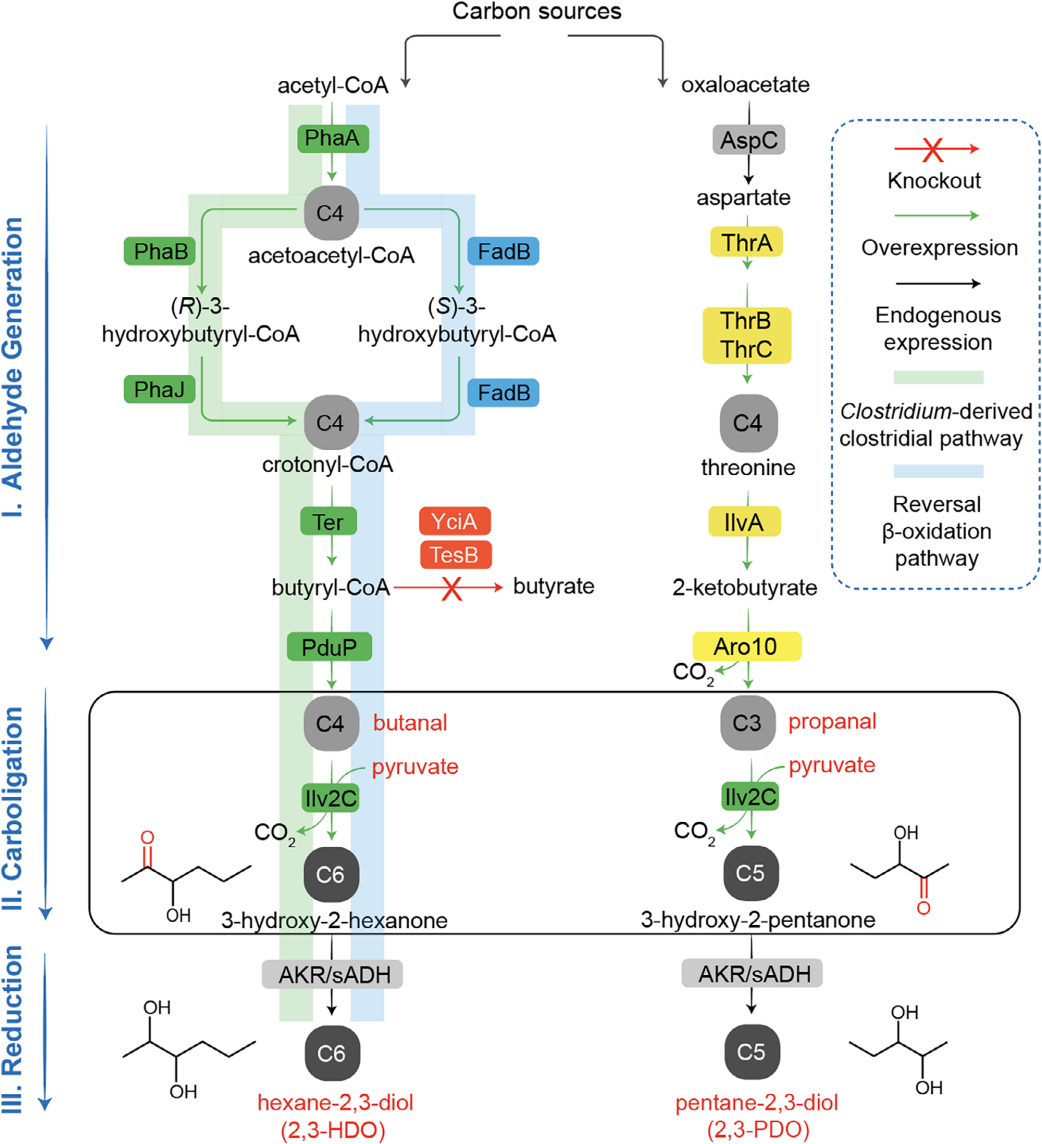
Schematic diagram of metabolic routes for the synthesis of linear chain β, γ‐diols. Synthetic pathways of hexane‐2,3‐diol and pentane‐2,3‐diol are divided into three parts: I. metabolic route for synthesis of butanal and propanal; II. Ilv2C carboligase mediated condensation of aldehyde with pyruvate to form the corresponding hydroxyketones; III. reduction of hydroxyketones to β,γ‐diols by endogenous aldo‐keto reductase (AKR) or secondary alcohol dehydrogenase (sADH). PhaA: Acetyl‐CoA acetyltransferase; PhaB: acetoacetyl‐CoA reductase; PhaJ: *R*‐specific crotonase; FadB: 3‐hydroxyacyl‐CoA dehydrogenase and enoyl‐CoA hydratase; Ter: trans‐enoyl‐CoA reductase; PduP: CoA‐acylating aldehyde dehydrogenase; Ilv2C: acetolactate synthase; AspC: aspartate aminotransferase; ThrA: aspartokinase I; ThrB: homoserine kinase; ThrC: threonine synthase; IlvA: threonine deaminase; Aro10: phenylpyruvate decarboxylase.

Recently, our group developed a microbial platform of branched‐chain β,γ‐diols from amino acid metabolism [24], which takes advantage of acetohydroxyacid synthase (AHAS)‐mediated recursive carbonation cycle to achieve the synthesis of 4‐methylpentane‐2,3‐diol, 5‐methylhexane‐2,3‐diol, and 4‐methylhexane‐2,3‐diol. In this study, we further expand the AHAS‐mediated carboligation for the synthesis of linear chain β,γ‐alkanediols in *E. coli* (Figure 1), namely, 2,3‐HDO and 2,3‐PDO. Although differing by only one carbon atom, the *de novo* biosynthesis of 2,3‐HDO and 2,3‐PDO relies on distinct aldehyde donor modules, demonstrating a modular and extensible biosynthetic platform for β,γ‐alkanediols. As shown in Figure 1, the upstream modules for butanal production involve two pathways for the production of butyryl‐CoA (a redesigned *Clostridium*‐derived clostridial pathway and an artificial reversal β‐oxidation pathway), followed by CoA‐acylating aldehyde dehydrogenase of PduP from *Salmonella enterica* [25, 26]. The butanal is condensed with pyruvate via the downstream module of Ilv2C (AHAS from *Saccharomyces cerevisiae*) followed by reduction by endogenous aldo‐keto reductases (AKRs) to achieve 2,3‐HDO production. By implementing a propanal biosynthetic route, 2,3‐PDO can also be realized from renewable feedstocks (Figure 1). Through systematic metabolic engineering and optimization of fermentation conditions, the engineered *E. coli* cells with expanded β,γ‐alkanediol biosynthetic pathways produced 152.2 mm (17.98 g/L) 2,3‐HDO in the fed‐batch bioreactor and 15.5 mm (1.61 g/L) 2,3‐PDO in shake flasks, which represents one of the pioneering works to realize linear chain β,γ‐alkanediols with carbon number ≥5 from artificial biosynthetic pathways.

## 2 Results

### 2.1 Establishment of 2,3‐HDO Biosynthesis via AHAS‐Mediated Carboligation

AHAS (EC 2.2.1.6) is a thiamine pyrophosphate (ThDP)‐dependent carbon‐carbon ligase that is ubiquitous in nature, including plants, fungi, and bacteria [27, 28, 29, 30]. The main physiological function of AHAS is to catalyze the carbon‐carbon ligation reaction between α‐keto acids such as pyruvate to produce acetohydroxyacids [31, 32, 33]. Based on our previous findings that AHAS‐mediated recursive carbonation cycle enables the synthesis of branched‐chain β,γ‐diols from branched‐chain amino acid (BCAA) metabolism [24], we reason that the substrate promiscuity of AHASs might be expanded to condense other linear chain aldehydes such as butanal with pyruvate.

**FIGURE 2:**
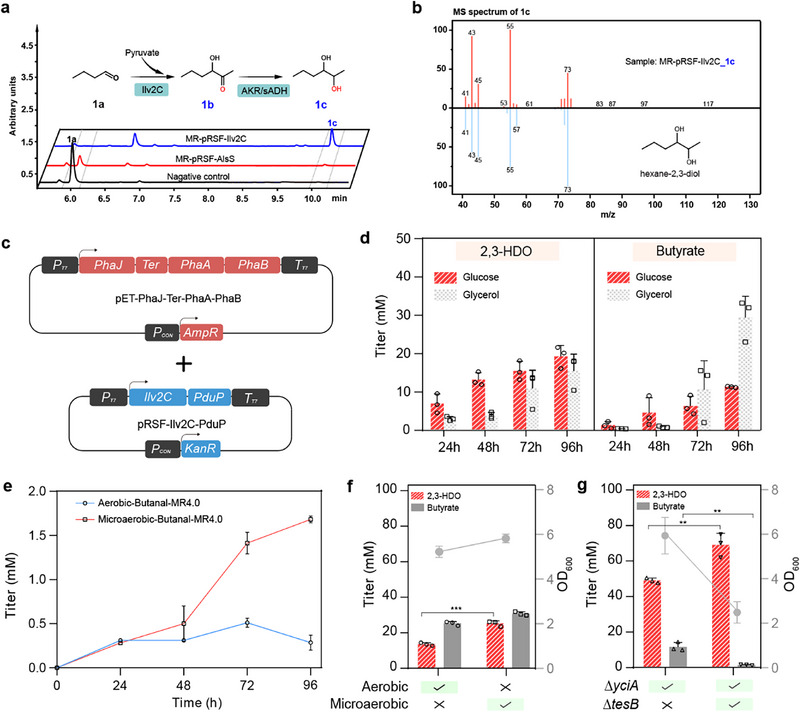
Functional identification of AHAS and engineering Clostridium‐derived clostridial pathway for hexane‐2,3‐diol biosynthesis. (a). Representative GC‐FID chromatogram results of carboligation of butanal and pyruvate catalyzed by MR‐AlsS and MR‐Ilv2c. Compound **1a** represents butanal, **1b** represents 3‐hydroxyhexan‐2‐one, and **1c** represents hexane‐2,3‐diol (2,3‐HDO). (b). Representative mass spectrum to validate the chemical structure of compound **1c** as compared to 2,3‐HDO from the NIST library. (c). The dual‐plasmid system for synthesizing 2,3‐HDO via the *Clostridium*‐derived clostridial pathway (the PhaB‐PhaJ route). (d). Exploring the carbon source preference for *de novo* synthesis of 2,3‐HDO. (e). Accumulation of butanal under aerobic and microaerobic conditions during *de novo* synthesis. (f). Profiles of 2,3‐HDO, butyrate, and biomass under aerobic and microaerobic conditions. The accumulation of 2,3‐HDO, butyrate, and biomass in the MR4.0 strain was compared at 96 h under aerobic and microaerobic conditions to evaluate the effect of oxygen availability on 2,3‐HDO production. (g). Effects of *yciA* and *tesB* deletions on 2,3‐HDO, butyrate, and biomass accumulation under microaerobic conditions. The average and standard deviation are obtained from three biological replicates. Statistical analysis was performed using an unpaired two‐tailed Student's *t*‐test, ^**^ indicates *p*‐value < 0.01, ^***^ indicates *p*‐value < 0.001.

In this study, we selected two potential AHASs to condense butanal with pyruvate, AlsS from *Bacillus subtilis*, and Ilv2C from *S. cerevisiae*, for 2,3‐HDO synthesis. AlsS has been extensively applied in metabolic engineering for 2,3‐butanediol production [34]. Based on previous observations, AlsS has activity toward aromatic aldehydes [35], representing one of the ThDP‐dependent enzymes with well‐recognized substrate diversity [36]. In comparison, Ilv2C from *S. cerevisiae* is less studied for the carboligation of pyruvate and different aldehydes. Upon confirming the successful expression as soluble fractions in *E. coli* (Figure S1), we prepared the whole cell biocatalysts for testing the activity of AHASs using butanal as the aldehyde donor. As shown in Figure 2a, after 24 h of whole‐cell catalysis, Ilv2C from *S. cerevisiae* rapidly consumed butanal (Figure 2a(1a), 6.437 min) to form a much higher level of product peak Figure 2a(1c) (9.961 min), as evidenced by GC‐MS analysis that the fragment pattern of Figure 2a(1c) is consistent with that of 2,3‐HDO from the mass spectrum library (Figure 2b). To further validate the product structure, the sample was treated with periodic acid, yielding the expected cleavage products, butanal and ethanal (Figure S2). The formation of these characteristic oxidation products further supports the assignment of the product as 2,3‐HDO. In comparison, AlsS from *B. subtilis* only consumed a small amount of butanal Figure 2a(1a) to form product Figure 2a (1c). These findings indicated that Ilv2C had a better carboligation activity than AlsS for condensing butanal with pyruvate, which would serve a key enzyme module for the subsequent *de novo* synthesis of 2,3‐HDO. In addition, we also observed that the activity of endogenous AKRs in *E. coli* was sufficient to completely convert compound Figure 2a(1b) to (1c) as no accumulation of intermediate Figure 2a(1b) was observed in the catalytic system based on gas chromatography analysis (Figure 2a).

Upon the successful demonstration of Ilv2C‐mediated effective carboligation of butanal with pyruvate, we next attempted to achieve *de novo* synthesis of 2,3‐HDO from renewable feedstocks. As described in Figure S3, the central metabolism typically produces an NADPH and NADH ratio at ∼0.2. As the native clostridial pathway extensively utilizes NADH to power the butanal biosynthesis, we therefore redesigned *Clostridium*‐derived clostridial pathway that contains alternative enzymes such as acetyl‐CoA acetyltransferase and acetoacetyl‐CoA reductase (PhaA and PhaB from *Ralstonia eutropha*) [37, 38, 39], *R*‐specific crotonase (PhaJ from *Aeromonas caviae*) [40] and trans‐enoyl‐CoA reductase (Ter from *Treponema denticola*) [41], which is used to produce butyryl‐CoA from the central metabolite of acetyl‐CoA with a more balanced NADH and NADPH utilization (Figure S3). Further combined with CoA‐acylating aldehyde dehydrogenase PduP from *S. enterica* enables the production of the butanal substrate for 2,3‐HDO synthesis by Ilv2C and endogenous AKRs (Figure 1). Notably, as compared to other butanal biosynthetic pathways that involve the decarboxylation of corresponding α‐keto acids from CimA‐LeuABCD [42], the utilization of the redesigned *Clostridium*‐derived clostridial pathway would more specifically produce 2,3‐HDO over other diols derived from α‐keto acids, which is expected to simplify the downstream separation and purification processes.

In order to maintain the high level of pyruvate for *de novo* 2,3‐HDO biosynthesis, we chose to use the previously constructed *E. coli* MR4.0 [24] with deletion of *pta*, *pflB*, *ldhA*, and *adhE* as the chassis, to minimize the conversion of pyruvate to fermentation byproducts such as lactate and ethanol. Upon introducing a dual‐plasmid system containing PhaJ‐Ter‐PhaAB and Ilv2C‐PduP (Figure 2c), the resulting strain enabled the production of approximately 19.2 mm 2,3‐HDO from 40 g/L glucose (Figure 2d). However, most of the butyryl‐CoA intermediate was diverted to the by‐product butyrate by endogenous acyl‐CoA thioesterases (TEs) [43, 44, 45]. In addition, by exploring the effect of glycerol as an alternative substrate on the production of 2,3‐HDO, it was found that glycerol was more inclined to the production of butyrate over 2,3‐HDO (Figure 2d). Therefore, glucose was considered as a more favorable substrate for subsequent fermentation experiments.

### 2.2 Metabolic Engineering of Clostridial Pathway‐Derived 2,3‐HDO Biosynthesis

In microbial fermentation, it is reported that self‐induced anaerobiosis fermentation may promote aldehyde accumulation by reducing gas exchange and maintaining a confined environment [46]. As shown in Figure S4, during external supplementation of butanal, the *E. coli* cell culture did retain better under microaerobic conditions than that of aerobic conditions. Further *de novo* synthesis of butanal with expressing PhaJ‐Ter‐PhaAB and PduP also confirmed that the microaerobic butanal level was accumulated 5.8‐fold higher that of aerobic conditions at 96 h (Figure 2e). Similarly, a comparison of aerobic and microaerobic fermentation in 2,3‐HDO based on the PhaB‐PhaJ route showed that microaerobic fermentation further increased the concentration of 2,3‐HDO to 24.9 mm, which was approximately 1.8‐fold improvement over that of aerobic fermentation (*p*‐value < 0.001) (Figure 2f). However, a large part of butyryl‐CoA was converted to butyrate (30.5 mm) under microaerobic fermentation (Figure 2f).

In *E. coli*, there are a number of acyl‐CoA Tes that may directly limit the flux of 2,3‐HDO by acting on acetyl‐CoA, acetoacetyl‐CoA, (*R*)‐3‐hydroxybutyryl‐CoA, crotonyl‐CoA, and butyryl‐CoA [16, 47]. In particular, TEs encoded by *tesB* and *yciA* have been used to boost the production of 3‐hydroxybutyrate [43, 44], and medium‐chain fatty acids [47]. Therefore, we chose to delete the dominant acyl‐Coa TEs of TesB and YciA (Figure S5), to reduce the metabolic flux loss toward butyrate. As shown in Figure 2g, it was found that ∆*yciA* and ∆*yciA*∆*tesB* substantially reduced the accumulation of butyrate to 10.9 mM and 0.8 mM (*p*‐value < 0.01), respectively. Meanwhile, the titer of 2,3‐HPO was further increased to 68 mm upon deletion of *yciA* and *tesB* (*p*‐value < 0.01) (Figure 2g). However, MR4.0∆*yciA*∆*tesB* resulted in a substantial reduction in biomass accumulation (Figure 2g). As butyrate and butanal share the same substrate of butyryl‐CoA, there might be an accumulation of butyryl‐CoA to a toxic level that would deplete the CoA moiety for other essential biochemical reactions such as acetyl‐CoA in energy metabolism.

### 2.3 Reversal β‐Oxidation Pathway Derived 2,3‐HDO Biosynthesis with Enhanced Productivity

**FIGURE 3:**
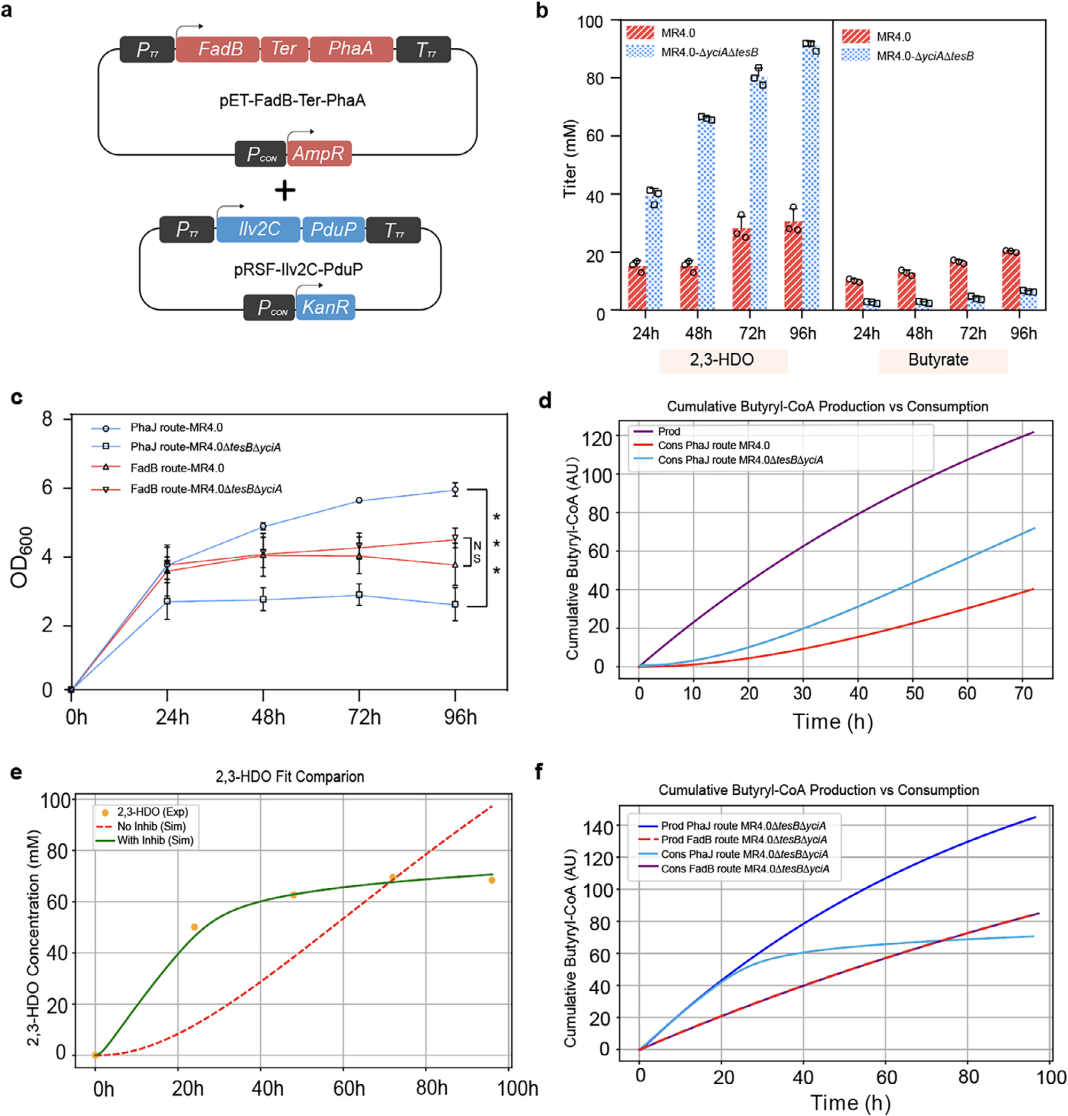
Engineering reversal β‐oxidation pathway for hexane‐2,3‐diol biosynthesis. (a). The dual‐plasmid system for synthesizing 2,3‐HDO via the reversal β‐oxidation pathway (the FadB route). (b). Profiles of 2,3‐HDO and butyrate under microaerobic conditions via the reversal β‐oxidation pathway. (c). Changes in biomass accumulation after knockout of thioesterases (YciA and TesB) in the PhaB‐PhaJ route vs. the FadB route. (d). Ordinary Differential Equation (ODE) model to fit MR4.0 and MR4.0∆*yicA*∆*tesB* for total production (Prod) and consumption (Cons) of butyryl‐CoA. (e). ODE Model with and without substrate inhibition model to fit MR4.0∆*yicA*∆*tesB* for 2,3‐HDO production. (f). Comparison of the rates of total synthesis and consumption of butyryl‐CoA by the PhaB‐PhaJ route vs. the FadB route. The average and standard deviation are obtained from three biological replicates. Statistical analysis was performed using unpaired unpaired two‐tailed Student's *t*‐test, NS indicates *p*‐value >0.05, ^***^ indicates *p*‐value < 0.001.

In the reversal β‐oxidation (rBOX) pathway, the biochemical properties of these enzymes allow for flexible precursor inputs and a wide variety of termination pathways to customize the length and class of the product chain [48, 49, 50], providing excellent flexibility and combinatorial capabilities for the production of a wide range of aliphatic aldehydes [51]. Therefore, the introduction of the rBOX‐based pathway for the production of butanal may provide insights for the subsequent production of diverse aldehyde substrates. Next, we introduced a butanal biosynthetic module that relies on the artificial rBOX pathway of the FadB route as depicted in Figure 1, which was also demonstrated in cyanobacteria [52]. After transferring this FadB‐mediated biosynthetic pathway (FadB‐Ter‐PhaA and Ilv2C‐PduP as shown in Figure 3a) into the MR4.0 strain, the 2,3‐HDO production reached a level of 30.5 mm under microaerobic conditions, with a byproduct butyrate level of 20.3 mm (Figure 3b). Further shake flask experiments using the MR4.0∆*yciA*∆*tesB* revealed that butyrate was reduced to 6.6 mm, whereas the titer of 2,3‐HDO was substantially increased to 91.1 mm (10.76 g/L, 0.269 g/g, 0.112 g/L/h). Surprisingly, we found that no obvious change of biomass accumulation occurred for the FadB‐mediated 2,3‐HDO biosynthetic route in MR4.0∆*yciA*∆*tesB* (Figure 3c), indicating that butyryl‐CoA might be efficiently converted to butanal by PduP without accumulating to a toxic level. According to the reported *K*m value of PduP is 0.87 µm [37], and the calculation of the relative rate based on the Michaelis‐Menten equation, the reaction rate of PduP can reach 98% of the maximum rate when the concentration of butyryl‐CoA reaches 4.35 mm (Figure S6). However, compared to the steady increase in 2,3‐HDO titer of the FadB route, the PhaB‐PhaJ route was characterized by a rapid accumulation compared to the FadB route before 24 h and a slower increase in 2,3‐HDO yield after 48 h (Figure 3b; Figure S7). Combined with the changes in biomass accumulation, we speculated that the lower 2,3‐HDO yield of the PhaB‐PhaJ route may be caused by substrate inhibition of butyryl‐CoA accumulation.

Ordinary differential equation (ODE) models are commonly used to fit expression levels of products that are difficult to detect in biological systems [53]. To verify our hypothesis, we simulated the total production and consumption of butyryl‐CoA by introducing an ODE model for strain MR4.0 and strain MR4.0∆*yciA*∆*tesB*. The fitting results revealed that the cumulative amount of butyryl‐CoA increased by 61.4% after ∆*yciA*∆*tesB* (Figure 3d). To further verify whether there is a substrate inhibition involved in PduP, we introduced a substrate inhibition kinetic model to fit the production of 2,3‐HDO. Akaike Informativeness Criterion (AIC) is a measure of the goodness of fit and complexity of a model through the residual sum of squares (RSS) of the model and the number of free parameters that need to be optimized when fitting the model. It was found that the substrate inhibition kinetic model fit the experimental data better compared to the normal model without substrate inhibition (normal model without inhibition: RSS = 51.0431, AIC = 13.6162, model with inhibition: RSS = 0.3964, AIC = −8.6733) (Figure 3e). Incorporation of a substrate inhibition term yielded a fitted *K*m of 0.87 µm and a *K*i of 2.60 mm, indicating high catalytic affinity toward butyryl‐CoA at low concentrations but pronounced inhibition at elevated levels. This kinetic feature explains why excessive butyryl‐CoA accumulation fails to enhance, and instead limits, 2,3‐HDO production.

To further validate that the FadB route helps to release the inhibitory effect of butyryl‐CoA on PduP. We also simulated the total production and consumption of butyryl‐CoA in MR4.0∆*yciA*∆*tesB* with the FadB route through the substrate inhibition kinetic with the ODE model. Based on the ODE model as shown in Figure 3f, the rate of butyryl‐CoA production by the FadB route was much slower, thereby relieving the substrate inhibition on PduP to better utilize butyryl‐CoA. These findings clearly suggested that the FadB route was more flux balanced toward 2,3‐HDO production than that of the PhaB‐PhaJ route in *E. coli*. Considering that the accumulation levels of butanal and butanol were too low to impose measurable toxicity (Figure S8), greater attention should be paid to enzyme‐level regulation to prevent the accumulation of specific toxic intermediates, particularly butyryl‐CoA, by ensuring a balanced flux between upstream precursor supply and downstream consumption.

### 2.4 *De Novo* Synthesis of 2,3‐PDO via AHAS‐Mediated Carboligation

**FIGURE 4:**
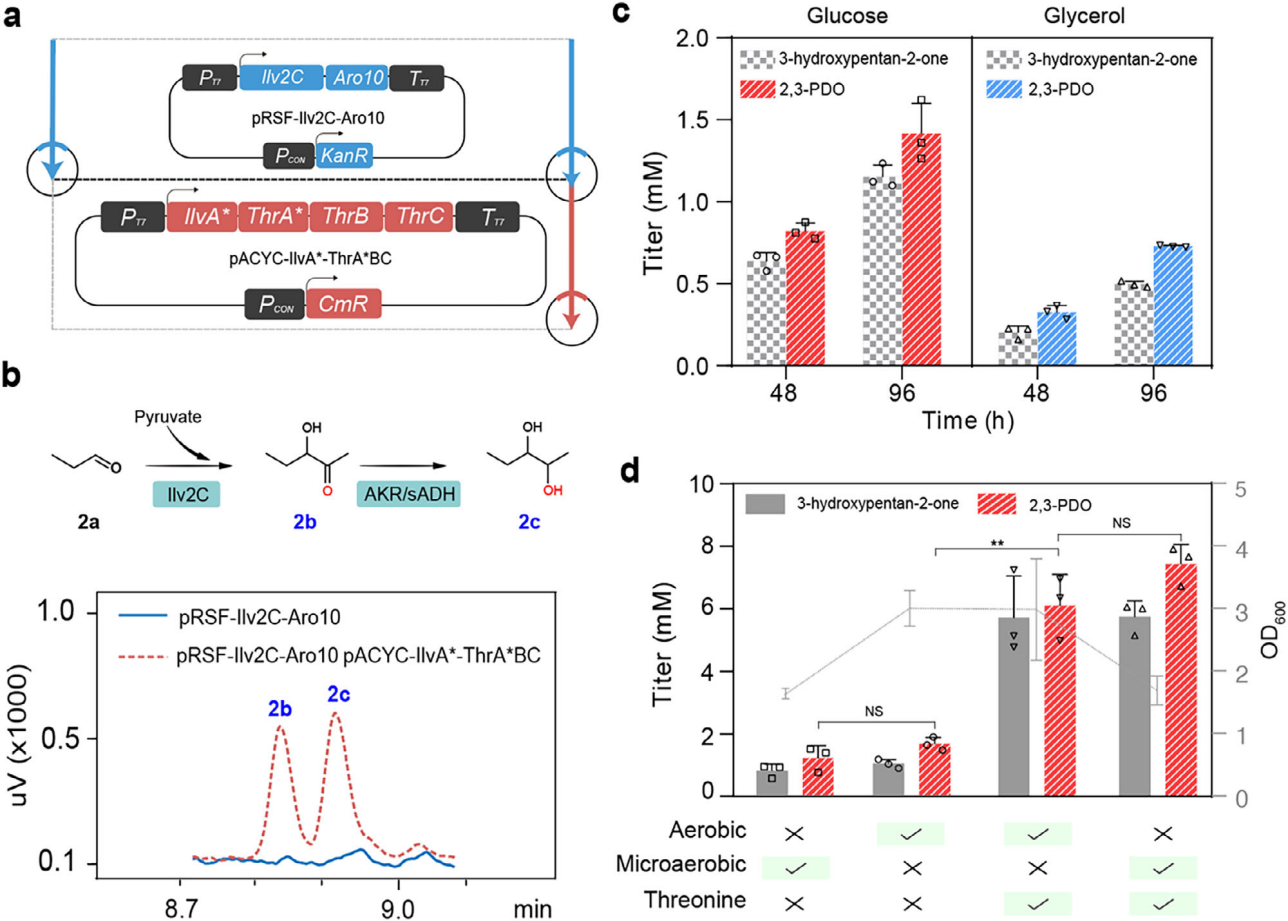
*De novo* synthesis of pentane‐2,3‐diol from L‐threonine metabolism. (a). The dual‐plasmid system for synthesizing pentane‐2,3‐diol (2,3‐PDO) from L‐threonine metabolism. (b). Comparison of the effect of L‐threonine‐biosynthetic module (IlvA^*^‐ThrA^*^BC) on *de novo* synthesis of 2,3‐PDO. IlvA^*^ (L447F, L451A) and ThrA^*^(S345F) are mutated enzymes with a feedback‐resistant feature. (c). Exploring the carbon source preference for *de novo* synthesis of 2,3‐PDO. (d). Effects of different fermentation conditions on biomass accumulation and 2,3‐PDO production. The average and standard deviation are obtained from three biological replicates. Statistical analysis was performed using an unpaired two‐tailed Student's *t*‐test, NS indicates *p*‐value > 0.05, ^**^ indicates *p*‐value < 0.01.

To further expand linear chain β,γ‐alkanediols, we attempted to establish a propanal synthetic module for 2,3‐PDO biosynthesis. We first examined PduP‐mediated release of propanal from propionyl‐CoA from the Sleeping Beauty mutase (Sbm) pathway [54] (Figure S9). Although the Sbm pathway has been used for high‐level production of propionate (reaching 30.9 g/L) [55], we found that Sbm‐coupled with PduP was not an efficient route for propanal production, as the addition of 3 g/L succinic acid to increase the supply of succinyl‐CoA could only slightly improve the propanal formation (Figure S9). Propanol biosynthesis from the L‐threonine synthetic pathway in *E. coli* [56] is well established, we therefore constructed a dual module system (IlvA^*^‐ThrA^*^BC and Ilv2C‐Aro10) to realize 2,3‐PDO biosynthesis from L‐threonine metabolism (Figure 4a). The first module contains a feedback resistant version of IlvA^*^ (L447F, L451A) coupled with ThrA^*^ (S345F) BC to strengthen 2‐ketobutyrate production [57]. The second module contains Ilv2C and a decarboxylase (Aro10 from *S. cerevisiae*), whereas 2‐ketobutyrate can decarboxylate into propanal to serve 2,3‐PDO production (Figure 1). As shown in Figure 4b, by expressing Ilv2C‐Aro10 in MR4.0, the engineered *E. coli* could not generate detectable amount for compound Figure 4b(2b) (3‐hydroxypentan‐2‐one, 3‐H‐P‐one) and compound Figure 4b(2c) (2,3‐PDO) due to insufficient accumulation of 2‐ketobutyrate (Figure 4b), whereas Ilv2C‐Aro10 was clearly functional as other branched diols were detected (Figure S10). Upon additional expression of IlvA^*^‐ThrA^*^BC, the formation of Figure 4b(2b) (8.821 min) and Figure 4b(2c) (8.908 min) could be detected by GC analysis (Figure 4a), which was further confirmed by mass spectrum analysis (Figure S11). To further validate the product structure, the sample was treated with periodic acid, yielding the expected cleavage products, propanal and ethanal (Figure S12). The formation of these characteristic oxidation products further supports the assignment of the product as 2,3‐PDO. The coexistence of intermediate compound Figure 4b(2b) indicated that the endogenous AKRs in *E. coli* poorly recognized this substrate for diol formation. The 2,3‐PDO reached <1.5 mm at 96 h cultivation under aerobic conditions (Figure 4c).

We also attempted to investigate the effect of changing the carbon source to glycerol on 2,3‐PDO production, but glycerol could not improve the production of 2,3‐PDO (Figure 4c). To reduce propanal vaporization under aerobic conditions (Figure S4), we employed microaerobic fermentation. However, 2,3‐PDO and its intermediate 3‐H‐P‐one did not increase, likely due to reduced biomass (Figure 4d). The addition of L‐threonine (3 g/L) improved the precursor availability, thereby increasing the microaerobic production of 2,3‐PDO to 7.5 mm (*p*‐value < 0.01) (Figure 4d). L‐Threonine addition also increased the proportion of linear‐chain diol containing the hydroxyketone to 36.45% (Figure S10).

### 2.5 Debottlenecking the Rate‐Limiting Step of 2,3‐PDO Biosynthesis

**FIGURE 5:**
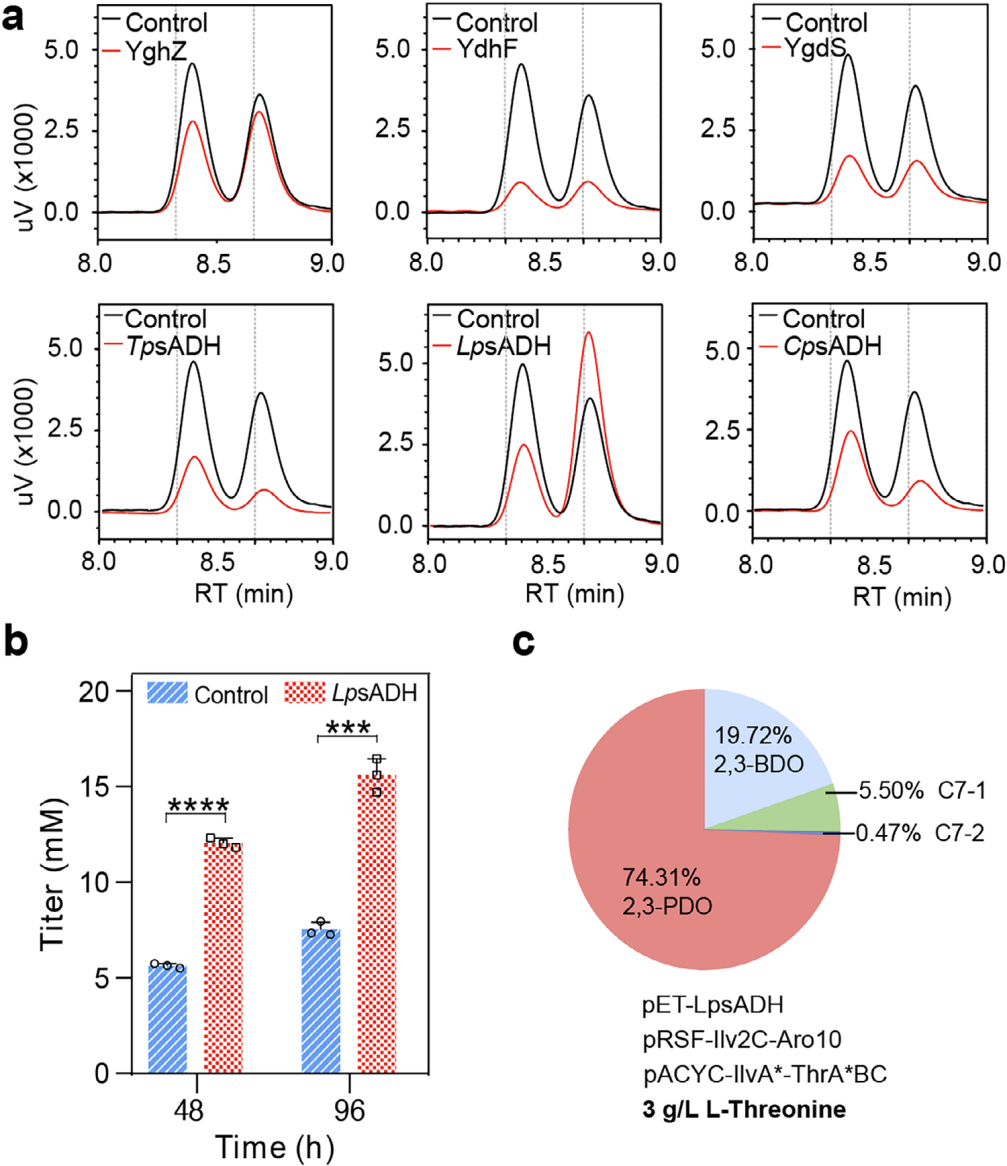
Debottlenecking the insufficient conversion of 3‐hydroxypentan‐2‐one to pentane‐2,3‐diol. (a). Comparison of the reduction of 3‐hydroxypentan‐2‐one by different endogenous AKRs and exogenous sADHs. YghZ, YdjG, and YdhF are endogenous AKRs from *E. coli*. *Tb*sADH, sADH from *T. brockii*; *Cp*sASH, sADH from *C. parapsilosis*; *Lp*sADH, sADH from *L. paseudomesenteroides*. (b). 2,3‐PDO production upon inducing *Lp*sADH. (c). Diol distribution profile upon inducing *Lp*sADH. The fermentation for 2,3‐PDO production was conducted in modified M9 supplemented with 3 g/L L‐threonine. The average and standard deviation are obtained from three biological replicates. Statistical analysis was performed using an unpaired two‐tailed Student's *t*‐test, ^***^ indicates *p*‐value < 0.001, ^****^ indicates *p*‐value < 0.0001.

Since L‐threonine is a relatively cheap and renewable substance, the limiting activity of endogenous AKRs toward 3‐H‐P‐one remains as a key bottleneck for high‐level 2,3‐PDO synthesis. To further address the insufficient activity of endogenous AKRs, we examined three previously reported AKRs (YghZ, YdjG, and YdhF) with improved diol synthesis [24]. Besides, we also chose several secondary alcohol dehydrogenases (sADHs) with reported activity toward the synthesis 2,3‐BDO [58], namely, *Tb*sADH from *Thermoanaerobacter brockii*, *Cp*sASH from *Candida parapsilosis*, *Lp*sADH from *Leuconostoc paseudomesenteroides*. As shown in Figure 5a, after shake tube screening, we identified that only sADH from *L. paseudomesenteroides* (*Lp*sADH) had a noticeable improvement on 2,3‐PDO with reduced proportion of 3‐H‐P‐one, whereas the remaining tested enzymes reduced levels of both 2,3‐PDO and 3‐H‐P‐one for unknown reasons. Further scale‐up in shake‐flasks revealed that *Lp*sADH could improve the 2,3‐PDO to 15.5 mm (1.61 g/L, 0.040 g/g, 0.0168 g/L/h) (Figure 5b), which was 2‐fold higher than that of its parental strain without *Lp*sADH (*p*‐value < 0.001). The distribution profile of diols also shifted toward 2,3‐PDO as the dominant product (Figure 5c), with additional 2,3‐BDO as the main byproduct. These findings suggested that *Lp*sADH could divert the flux from BCAA metabolism to favor 2,3‐BDO instead of other C6/C7 diols, indicating that the native metabolism of *E. coli* could spontaneously form (*S*)‐acetoin as previously observed in cyanobacteria [58].

### 2.6 Optimization of 2,3‐Hexanediol Production in Fed‐Batch Bioreactors

**FIGURE 6:**
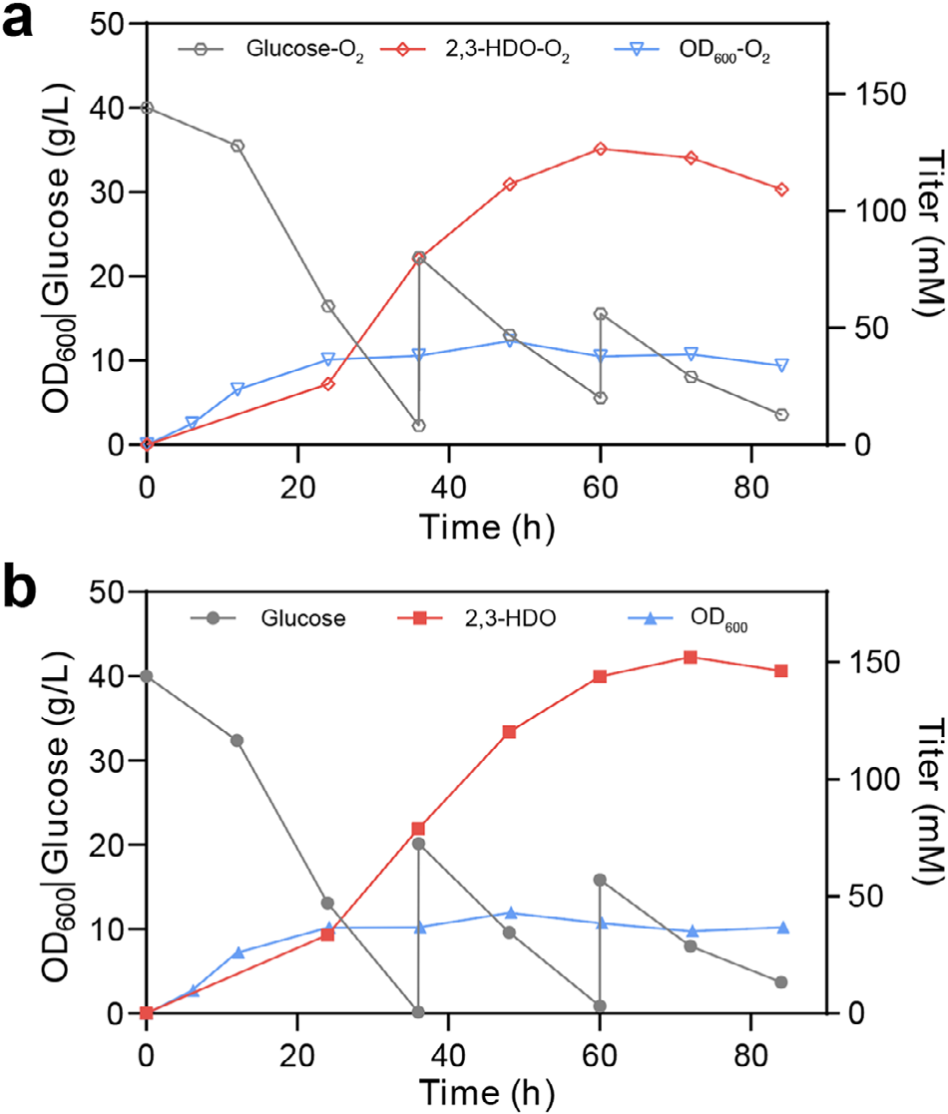
Time‐course fermentation profiles of 2,3‐hexanediol production in fed‐batch bioreactors. Profiles of glucose, biomass, and 2,3‐HDO under constant aeration at 20% O_2_ throughout the entire fermentation process (a) and two‐stage aeration strategy with microaerobic conditions (b) are presented. A 5‐L stirred‐tank bioreactor with 2 L modified M9 medium was used to evaluate the 2,3‐HDO production under controlled conditions. A two‐stage aeration strategy was set as following: 20% O_2_ was maintained during the first 24 h for rapid cell growth, followed by reduced aeration at 5% O_2_ to enhance carbon flux toward 2,3‐HDO biosynthesis.

Lastly, we prioritized 2,3‐HDO as the target product for scale‐up studies because it can be obtained with a higher titer in shake flasks, thereby conferring greater potential for industrial applications. Time‐course fermentations of 2,3‐HDO under two aeration strategies are presented in Figure 6. Under constant aeration at 20% O_2_, the 2,3‐HDO concentration increased to 126.6 mm (15.0 g/L) at 60 h, accompanied by the consumption of 54.46 g/L glucose. This corresponded to a product yield of 0.275 g/g and a volumetric productivity of 0.25 g/L/h; extending the cultivation beyond 60 h did not result in any further increase in titer.

In contrast, a two‐stage aeration strategy (20% O_2_ for the first 24 h, followed by 5% O_2_) improved carbon conversion efficiency. At 60 h, the culture achieved 143.9 mm (17.0 g/L) 2,3‐HDO with 59.15 g/L glucose consumed, corresponding to a yield of 0.287 g/g and a volumetric productivity of 0.28 g/L/h. Although a slightly higher titer of 152.2 mm was observed at 72 h, the incremental increase after 60 h was minimal, indicating that the majority of product synthesis occurred within the first 60 h under the two‐stage aeration regime. During scale‐up fermentation, a pronounced product‐associated odor was detected, suggesting partial volatilization of 2,3‐HDO into the exhaust gas. To mitigate volatile loss of 2,3‐HDO, future installation of a condenser on the exhaust line of the bioreactor should be considered during the process optimization [59].

## 3 Discussion

In this work, we successfully expanded the microbial synthesis of linear chain β,γ‐alkanediols to 2,3‐HDO and 2,3‐PDO via artificially designed biosynthetic pathways. Our work focused on exploring chain elongation on linear chain aldehyde by C+2 mediated by AHAS. Based on our results, we found that microaerobic condition generally favor the β,γ‐alkanediol biosynthesis by preventing the vaporization loss of volatile aldehydes. During the investigation of 2,3‐HDO biosynthesis, we found that the redesigned PhaB‐PhaJ route would cause substrate inhibition to PduP and led to the accumulation of butyryl‐CoA at a toxic level that reduces the cell growth (Figure 3c), which was further confirmed by ODE simulation results (Figure 3d). On the other side, the reversal β‐oxidation pathway with a slow butyryl‐CoA production rate could maintain a balanced metabolic flux for more efficient synthesis of 2,3‐HDO. Both experimental fermentation data and model simulations suggested that the PhaB‐PhaJ route exhibits a higher intrinsic rate of butyryl‐CoA production; however, this advantage is offset by pronounced substrate inhibition of PduP, which restricts effective downstream conversion. Although intermediate accumulation could in principle be mitigated through extensive regulatory or enzyme engineering strategies, such interventions entail substantial experimental complexity. Consequently, the FadB route provides a more practical and economically favorable solution for establishing an efficient 2,3‐HDO biosynthetic platform. Considering the native MhpF from *E. coli* has been reported with a good activity toward 1‐butanol overproduction [47], it might be introduced to alleviate the substrate inhibition occurred at the PduP step.

During the production process of 2,3‐PDO, it was found that the abundance of 2‐ketobutyrate from L‐threonine remained as the main bottleneck that limits efficient production of 2,3‐PDO. As shown in Figure 4d, supplementation with 3 g/L L‐threonine markedly increased 2,3‐PDO titers, indicating that endogenous L‐threonine supply was insufficient to fully support downstream biosynthesis. As a previous study reported that extensive metabolic engineering modifications of *E. coli* could achieve a titer of 170.3 g/L L‐threonine [60, 61], such strategies could be straightforward for improving L‐threonine in our 2,3‐PDO synthesis system. Alternatively, other microorganism hosts such as *Corynebacterium glutamicum* with high level of L‐threonine might be implemented for the subsequent production to 2,3‐PDO [61, 62]. As the essential BCAA‐related pathway shares similar α‐keto acids that can be readily decarboxylated to aldehydes, it requires dynamic regulation of the BCAA metabolism to increase the specific production of 2,3‐PDO over other branched‐chain diols. For instance, light‐controlled CRISPRi knocking down at key branching points such as *IlvC* or *IlvD* would ensure biomass accumulation during the growth phase, while conditionally repressed during the 2,3‐PDO production stage [63]. Although the propionate titer from Sbm pathway was reported to reach 30.9 g/L [55], we failed to divert propionyl‐CoA from Sbm pathway to produce propanal at a high titer. Considering that the catalytic activity of PduP toward propionyl‐CoA is higher than that of butyryl‐CoA [40], the problem should be attributing to some unknown variations during the engineering Sbm pathway and extensive works are required to redistribute of metabolic flux to propanal by knocking out genes like *gldA*, *dhaK*, *iclR*, and *sdhA* [54, 55]. Through extensive screening of endogenous AKRs and exogenous sADHs, we identified *Lp*sADH with improved conversion of 3‐H‐P‐one to 2,3‐PDO (Figure 5a), but there was still a substantial amount of 3‐H‐P‐one being accumulated. To fully realize the biosynthetic potential of 2,3‐PDO, future efforts are required to mine and engineer efficient AKRs or sADHs to actively and specifically accept 3‐H‐P‐one as the preferred substrate.

In summary, through systematic metabolic engineering and optimization of fermentation conditions, the engineered *E. coli* cells with expanded β,γ‐alkanediol biosynthetic pathways produced 152.2 mm (17.98 g/L) 2,3‐HDO in the fed‐batch bioreactor and15.5 mm (1.61 g/L) 2,3‐PDO in shake flasks, respectively. Compared with existing microbial diol production technologies, clear differences in technological maturity can be observed between established α,β‐diol platforms and the emerging β,γ‐diol platform (Table S1). Diol products such as 2,3‐BDO and 1,4‐BDO have been extensively investigated and have benefited from continuous metabolic engineering and pathway optimization, resulting in high production metrics at laboratory, pilot‐scale, and industrial levels. In contrast, the microbial biosynthetic pathways for β,γ‐alkanediol (2,3‐HDO and 2,3‐PDO) are reported for the first time in this study. Although the achieved yields reflect only an early developmental stage of microbial β,γ‐diol production, our work establishes a foundation for further yield improvement through enzyme engineering and fermentation optimization. With the advancement of synthetic biology, alternative cheap resources such as agricultural wastes including cellulose and lignin [64, 65] may provide a sustainable and economic route for β,γ‐alkanediol productions in various aldehyde‐accumulating microorganisms [66].

## 4 Methods

### 4.1 General Reagents

*E. coli* Top10 was used for the routine cloning of plasmids. *E. coli* MR4.0, a derivative of *E. coli* RARE (∆*dkgB*, ∆*yeaE*, ∆*dkgA*, ∆*yqhC*, ∆*yqhD*, ∆*yahK*, ∆*yjgB*) [67], was used for diol biosynthesis. All restriction enzymes, T4 ligase and DNA polymerase were purchased from New England Biolabs (Ipswich, MA, USA). All gel extraction kits, and plasmid DNA extraction kits were obtained from BioFlux (Shanghai, China). Chemicals used in this study were purchased from Aladdin and Macklin (Shanghai, China) unless otherwise stated.

Luria‐Bertani (LB) medium (10 g/L tryptone, 5 g/L yeast extract, and 10 g/L NaCl) was used for cultivating the *E. coli* cells with corresponding antibiotics (ampicillin 100 µg/mL, kanamycin 50 µg/mL, and chloramphenicol 34 µg/mL). For fermentative production diols, the modified M9 medium (4.27 g/L Na_2_HPO_4_·12H_2_O, 0.75 g/L KH_2_PO_4_, 0.25 g/L NH_4_Cl, 0.125 g/L NaCl, 11.1 µg/L CaCl_2_, 240.7 mg/L MgSO_4_, 50 mg/L EDTA, 8.3 mg/L FeCl_3_·6H_2_O, 0.84 mg/L ZnCl_2_, 0.13 mg/L CuCl_2_·2H_2_O, 0.1 mg/L CoCl_2_·6H_2_O, 0.1 mg/L H_3_BO_3_, 16 mg/L MnCl_2_·6H_2_O, 0.3 mg/L Na_2_MoO_4_·4H_2_O, 5 mg/L thiamin hydrochloride, 10 mg/L nicotinic acid, 0.1 mg/L biotin, 5 g/L yeast extract, 10 g/L tryptone, 40 g/L glucose or 40 g/L glycerol) was used.

### 4.2 Plasmids Construction

Genes encoding acetyl‐CoA acetyltransferase (*PhaA*) and acetoacetyl‐CoA reductase (*PhaB*) were PCR amplified from plasmid pBHR68 [68]. Genes encoding *R*‐specific crotonase (*PhaJ*, GenBank: WP_168235189.1) and trans‐enoyl‐CoA reductase (*Ter*, Gene ID: 4GGO_A) were codon‐optimized and synthesized by GenScript (Nanjing, Jiangsu, China). Genes encoding 3‐Hydroxyacyl‐CoA dehydrogenase and enoyl‐CoA hydratase (*FadB*, GenBank: EOK1910949.1), aspartokinase I (*ThrA*, GenBank: ABI99494.1), momoserine kinase (*ThrB*, GenBank: WP_112031210.1), threonine synthase (*ThrC*, GenBank: EOT4177360.1), and threonine deaminase (*IlvA*, GenBank: NP_418220.1) were cloned from the genomic DNA of *E. coli* MG1655. Genes encoding acetolactate synthase (*Ilv2C*, GenBank: NM_001182608.1) and phenylpyruvate decarboxylase (*Aro10*, GenBank: NM_001180688.3) were cloned from the genomic DNA of *S. cerevisiae* BY4741. Gene encoding acetolactate synthase (*AlsS*, GenBank: NP_391482.2) were cloned from the genomic DNA of *B. subtilis*. Gene encoding CoA‐acylating aldehyde dehydrogenase (*PduP*, GenBank: WP_061376584.1) was PCR amplified from the genomic DNA of *S. enterica*. Endogenous AKR‐related genes *YghZ* (GenBank: WGB75712.1), *YdjG* (GenBank: WP_201476341.1), and *YdhF* (GenBank: WP_313708273.1) were PCR amplified from the genomic DNA of *E. coli* MG1655. Heterologous sADH‐related genes of *CpsADH* (GenBank: 7DLM_A), *TbsADH* (GenBank: WP_041589967.1), and *LpsADH* (GenBank: WP_084058241.1) were codon‐optimized and synthesized by GenScript (Nanjing, Jiangsu, China).

The primers used in this study are listed in Table S2. *Ilv2C* and *PduP*/*Aro10* were inserted into the pRSFDuet1 between *Bam*HI/*Xho*I sites to obtain the plasmid pRSF‐Ilv2C‐PduP and pRSF‐Ilv2C/Aro10. The pETDuet1‐PhaJ‐Ter, pET‐TbsADH/ CpsADH/LpsADH were codon optimized and directly synthesized by GenScript (Nanjing, Jiangsu, China). *PhaAB* from plasmid pBHR68 was inserted into the pETDuet1‐PhaJ‐Ter plasmid between *Bgl*II‐*Xho*I sites. *Ter‐PhaA* was ligated with the *FadB* fragment into the *Bam*HI/*Xho*I site of pETDuet1 to obtain the plasmid pET‐FadB‐Ter‐PhaA. The overlapping PCR obtained *IlvA* ^fbr^ (L447F, L451A) and *ThrA* ^fbr^ (S345F) *BC* fragments were ligated into the pACYCDuet1 between *Bam*HI/*Xho*I sites to obtain the plasmid pACYC‐IlvA^*^‐ThrA^*^BC. *YghZ*, *YdjG*, and *YdhF* were inserted into the pETDuet1 plasmid between *Bam*HI/*Xho*I sites. *TbsADH*, *CbsADH*, and *LpsADH* related sADH genes were inserted into the pETDuet1 plasmid between *Bam*HI/*Xho*I sites. All plasmids used in this study are provided in Table S3. All plasmids were transformed into *E. coli* MR4.0 [24], and the resulting strains used in this study are provided in Table S4.

### 4.3 Biocatalysis Procedures

Whole‐cell biotransformation was conducted as previously described [24, 69]. Colonies were first inoculated into liquid LB medium supplemented with appropriate antibiotics and incubated at 37°C at 250 rpm for 12 to 16 h to obtain a seed culture. The seed culture was inoculated at 1% (v/v) into Terrific Broth medium (24 g/L yeast extract, 12 g/L tryptone, 0.4% glycerol, 0.017 m KH_2_PO_4_, 0.072 m K_2_HPO_4_) were incubated at 37°C at 250 rpm to an OD_600_ of 0.6–0.8. Then, isopropyl‐β‐D‐thiogalactopyranoside (IPTG) was added to a final concentration of 0.5 mm, and the cell cultures were transferred to 20°C and 250 rpm conditions for induced expression of proteins for 16–18 h. Biocatalysis was carried out at 30°C and 250 rpm for 24 h with a 1 mL system containing 10 g/L glucose, 10 g cell dry weight (CDW)/L biocatalysts, substrates, and phosphate buffer (100 mm, pH 7.0). Control reactions were represented by samples collected at 0 h, before the addition of whole‐cell biocatalysts. Samples were exacted by organic solvent for analysis using gas chromatography equipped with a flame ionization detector (GC‐FID).

### 4.4 Fermentation Production of Diols

Colonies were inoculated into liquid LB medium containing the appropriate antibiotics and incubated at 37°C, 250 rpm for 12–16 h to prepare seed cultures. A 1% (v/v) seed culture was transferred into 20 mL of modified M9 medium for diol production. Cultures were first grown in a shaking incubator at 37°C and 250 rpm for 3 h. IPTG was then added to a final concentration of 100 µm when OD_600_ reached 0.6–1.0. Cells were subsequently incubated at 30°C for diol biosynthesis. Aerobic and microaerobic fermentations were conducted in 100 mL conical flasks containing 20 mL of culture medium. For aerobic conditions, flasks were sealed with a high‐temperature‐resistant tissue culture sealing membrane with aeration holes to allow sufficient gas exchange. For microaerobic conditions, flasks were tightly sealed by aluminum foil to restrict oxygen transfer.

A 2‐L scale‐up fermentation was conducted in a 5‐L stirred‐tank bioreactor to evaluate the 2,3‐HDO production under controlled conditions. 200 mL of seed culture was inoculated into 2 L modified M9 medium, corresponding to an inoculation ratio of 1% (v/v). The cultivation temperature was initially set to 37°C. After 12 h of fermentation, the cultivation temperature was shifted to 30°C, and IPTG was added to a final concentration of 200 µm. At the start of fermentation, the agitation speed and aeration rate were set to 300 rpm and 2 L/min, respectively, and subsequently adjusted to maintain the dissolved oxygen (DO) level at approximately 20%. For a microaerobic condition, the aeration rate was reduced to 0.5 L/min to achieve a lower DO level of approximately 5%. The glucose pulse feeding was initiated when the residual glucose concentration decreased below 10 g/L. Throughout the fermentation process, samples were collected at 12‐h intervals for the quantification of 2,3‐HDO production and residual glucose concentration.

Quantification of metabolites including diols, aldehydes, and α‐hydroxyketones was conducted using GC‐FID on a Nexis GC‐2030 system. Separation was achieved using an SH‐Rtx‐5 capillary column (25 m × 0.32 mm × 0.5 µm). Fermentation samples were extracted with ethyl acetate, while whole‐cell catalytic samples were extracted with dodecane. For analysis, 1 µL of the organic phase was injected in splitless mode with an injector temperature of 250°C. Nitrogen served as the carrier gas at a column flow rate of 1.0 mL/min. The oven temperature was programmed as follows: initial temperature of 40°C (held for 2 min), ramped at 15°C/min to 250°C, and held for 2 min. The FID detector was set at 350°C. Butanal and propanal standards were used to generate calibration curves. Due to the lack of commercially available diol standards, isomers such as 1,5‐pentanediol and 1,6‐hexanediol were used for plotting external standard curves. The concentrations of α‐hydroxyketones were calculated based on their corresponding diols.

For the quantitation of the residual glucose, 300 µl of fermentation broth was mixed with 300 µl of distilled and deionized water (ddH_2_O), centrifuged at 13500 rpm for 10 min, and the supernatant was filtered through a 0.22 µm filter to remove the residual cell pellets. The samples were next analyzed by high performance liquid chromatography (HPLC). A Shimadzu LC‐20A system equipped with a refractive index detector (RID) and an Aminex HPX‐87H organic acid analysis column (300 mm x 7.8 mm, 9 µm) was used. The column temperature was maintained at 50°C, ultrapure water containing 5 mM sulfuric acid was used as the mobile phase, and the flow rate was maintained at 0.5 mL/min.

Gas chromatography‐mass spectrometry (GC‐MS) analysis was performed using an Agilent 7977B MSD system to confirm the identities of metabolites such as diols and α‐hydroxyketones. The system was equipped with an Agilent HP‐5 ms Ultra Inert GC column (30 m × 250 µm × 0.25 µm). A 1 µL aliquot of each sample was injected via autosampler with a split ratio of 1:1. Helium served as the carrier gas at a flow rate of 1.0 mL/min. The oven temperature was initially set to 40°C (held for 2 min), then increased to 250°C at 15°C/min and held for 2 min. The mass spectrometer was operated in scan mode with a solvent delay of 3.5 min. The ion source and quadrupole temperatures were set to 230°C and 150°C, respectively. Metabolite identification was based on comparison of fragmentation patterns with reference spectra in the NIST 14 Mass Spectral Library.

### 4.5 Simulation Models

By defining the full reaction scheme and incorporating the experimental data points, the model enables simulation of the dynamic behaviors of all metabolites in the system, thereby facilitating a mechanistic understanding of pathway operation. The detailed ODE construction workflow is provided in Figure S13. To validate the accumulation of butyryl‐CoA in the PhaB‐PhaJ route, we fitted the production of hexane‐2,3‐diol (2,3‐HDO) under microaerobic conditions in the MR4.0 strain. To simplify the model, we integrated multiple reactions of glucose to butyryl‐CoA, and the reaction constant is *K*a1. The reaction is as follows:

glucose(40g/L)→butyryl−CoA

Butyryl‐CoA was transformed into butanal under the action of PduP, and the reaction constant is *K*m1. At the same time, butyryl‐CoA can also generate butyrate under the action of endogenous thioesterases, and the reaction constant is *K*a2. The reaction is as follows:

butyryl−CoA→butanal

butyryl−CoA→butyrate

Finally, 2,3‐HDO was generated from butanal under the action of Ilv2C, and the reaction constant is *K*a3. The reaction is as follows:

butanal→hexane−2,3−diol

To simulate the dynamics of butyryl‐CoA, we used ODEs to model the above reactions. The ODEs are given as follows:

$$\frac{d\left[glucose\right]}{dt}=-Ka1\left[glucose\right]$$

$$\begin{array}{cc} \frac{d\left[butyryl-CoA\right]}{dt} & =Ka1\left[glucose\right]-Km1\left[butyryl-CoA\right]\\ & \;-Ka2\left[butyryl-CoA\right] \end{array}$$

$$\frac{d\left[butanal\right]}{dt}=Km1\left[butyryl-CoA\right]-Ka3\left[butanal\right]$$

$$\frac{\left[butyrate\right]}{dt}=Ka2\left[butyryl-CoA\right]$$

$$\frac{d\left[hexane-2,3-diol\right]}{dt}=Ka3\left[butanal\right]$$

By substituting the experimental data points of butyrate and 2,3‐HDO from 24 to 96 h into the ODEs, the dynamic profiles of the corresponding compounds over this time period were simulated. To specifically examine the dynamics of butyryl‐CoA and butanal, the fitted kinetic constants *K*a1 and *K*a3 were fixed in the ODE model and applied to simulations of the MR4.0∆*tesB*∆*yciA* strain, reducing confounding effects arising from differences among chassis strains. Based on the above model, the reaction of depleting butyryl‐CoA into butyrate was removed. The ODEs are given as follows:

$$\frac{d\left[glucose\right]}{dt}=-Ka1\left[glucose\right]$$

$$\frac{d\left[butyryl-CoA\right]}{dt}=Ka1\left[glucose\right]-Km2\left[butyryl-CoA\right]$$

$$\frac{d\left[butanal\right]}{dt}=Km2\left[butyryl-CoA\right]\;-Ka3\left[butanal\right]$$

$$\frac{d\left[hexane-2,3-diol\right]}{dt}=Ka3\left[butanal\right]$$

After the model revealed the accumulation of butyryl‐CoA (Figure 3d), Michaelis–Menten kinetics: V =$\frac{Vmax\;*\;[S]}{Km+[S]\;}\;$ was used to predict the percentage of the reaction rate reaching Vmax at different substrate concentrations (V/Vmas =$\frac{\left[S\right]}{Km+[S]}\;$). According to the reported Km value of PduP [40], when the [S] = 50×Km (4350 µm), the rate is about 98% of Vmax (approaching saturation). Moreover, the model‐simulated increase in 2,3‐HDO production does not align with the nearly stagnant trend observed in the experimental data (Figure 3e), indicating the excessive accumulation of butyryl‐CoA in the PhaB‐PhaJ route group may lead to substrate inhibition of PduP.

In order to further verify the presence of substrate inhibition, we scored the dynamic changes of 2,3‐HDO fitted by the above ODE model (without substrate inhibition) and the ODE with substrate inhibition mode ($v=\frac{Vmax[S]}{Km+\left[s\right]+\frac{{\left[S\right]}^{2}}{Ki}}\;$).

In order to maintain the consistency of the model, the *K*a1, *K*a3, and *K*m of the above substrate‐free inhibition model were used. The ODEs with the substrate inhibition model are given as follows:

$$\frac{d\left[glucose\right]}{dt}=-Ka1\left[glucose\right]$$

$$\begin{array}{cc} & \frac{d\left[butyryl-CoA\right]}{dt}\\ & \;=Ka1\left[glucose\right]-\frac{Vmax\left[butyryl-CoA\right]}{Km+\left[butyryl-CoA\right]+\frac{{\left[butyryl-CoA\right]}^{2}}{Ki}} \end{array}$$

$$\begin{array}{cc} & \frac{d\left[butanal\right]}{dt}\\ & \;=\frac{Vmax\left[butyryl-CoA\right]}{Km+\left[butyryl-CoA\right]+\frac{{\left[butyryl-CoA\right]}^{2}}{Ki}}-Ka3\left[butanal\right] \end{array}$$

$$\frac{d\left[hexane-2,3-diol\right]}{dt}=Ka3\left[butanal\right]$$

The Residual Sum of Squares (RSS) is a measure of the difference between model predictions and experimental observations. The formula is as follows:

$$\mathrm{RSS}\;=\sum_{i=1}^{n}{\left(y_{i}^{\mathrm{obs}}-y_{i}^{\mathrm{pred}}\right)}^{2}$$

The $y_{i}^{\mathrm{obs}}$ is the experimental value at the i time point, the $y_{i}^{\mathrm{pred}}$ is the model predictive value at the i time point, and n is the number of data points.

The smaller the RSS is, the smaller the model fitting error is. However, RSS only focuses on the goodness of fit and does not consider the complexity of the model, which easily leads to overfitting. The Akaike Information Criterion (AIC) is a scoring system designed to balance between goodness of fit and model complexity.

$$\mathrm{AIC}\;=\;n\times \ln\left(\frac{\mathrm{RSS}}{n}\right)+2k$$

n: the number of observations (the number of data points), RSS: residual sum of squares, k: the number of adjustable parameters in the model. The smaller the AIC indicate the less and simpler the parameters used in the model while maintaining the accuracy. By comparison: normal model without inhibition: RSS = 51.0431, AIC = 13.6162; model with inhibition: RSS = 0.3964, AIC = 8.6733. The RSS and AIC of the ODE model with substrate inhibition are smaller than those of the ordinary ODE model, which indicates that PduP has the possibility of substrate inhibition.

The dynamic changes of butyryl‐CoA in the MR4.0∆*tesB*∆*yciA* strain were further compared between the FadB route and the PhaB‐PhaJ route. To maintain model consistency, the rate constant of the Ilv2C‐catalyzed carboligation reaction and the parameters describing substrate inhibition were kept consistent across simulations (fixed *K*a3, *V*m, *K*m, and *K*i), the ODEs with substrate inhibition model formulas are given as follows:

$$\frac{d\left[glucose\right]}{dt}=-Kb1\left[glucose\right]$$

$$\begin{array}{cc} & \frac{d\left[butyryl-CoA\right]}{dt}\\ & \;=Kb1\left[glucose\right]-\frac{Vmax\left[butyryl-CoA\right]}{Km+\left[butyryl-CoA\right]+\frac{{\left[butyryl-CoA\right]}^{2}}{Ki}} \end{array}$$

$$\begin{array}{cc} & \frac{d\left[butanal\right]}{dt}\\ & \;=\frac{Vmax\left[butyryl-CoA\right]}{Km+\left[butyryl-CoA\right]+\frac{{\left[butyryl-CoA\right]}^{2}}{Ki}}\;-Ka3\left[butanal\right] \end{array}$$

$$\frac{d\left[hexane-2,3-diol\right]}{dt}=Ka3\left[butanal\right]$$

The values of the reaction constants obtained through the process of fitting the experimental data can be found in Table S5. The entire model construction process and all calculation procedures are provided in the Supplementary Code File.

### 4.6 Quantitation and Statistical Analysis

Data were analyzed using GraphPad Prism version 9.0.1 and Excel Office. Statistical analysis was performed using unpaired two‐tailed Student's *t*‐test. All the statistical details of experiments can be found in the figure legends and results.

## Author Contributions

J.Y. conceived and designed the study; H.C., D.L., H.Y., and G.Y. performed the experiments and collected the data; Y.X. and Y.Z. assisted the experiments; H.C. and J.Y. interpreted the data and wrote the manuscript.

## Conflicts of Interest

The authors declare no conflicts of interest.

## Code Availability Statement

The process of model construction is described in detail in this paper, and the original code used to generate the results is available in the Supplementary Code File.

## Supporting information




**Supporting File**: advs74405‐sup‐0001‐SuppMat.docx.

## Data Availability

Data supporting the findings of this work are available within the paper and its Supplementary Information files. A reporting summary for this Article is available as a Supplementary Information file. Source data are provided with this paper.
