# Supplementary material for: Engineering New‐to‐Nature Biological Pathways for β,γ‐Alkanediol Synthesis
Source: Adv Sci (Weinh). 2026 Feb 12;13(23):e19742. doi: 10.1002/advs.202519742 (PMC13104072; doi:10.1002/advs.202519742)
Supplement: Supplementary file 1 — Supporting File: advs74405‐sup‐0001‐SuppMat.docx. [file ADVS-13-e19742-s001.docx]

**Engineering new-to-nature biological pathways for β,γ-alkanediol synthesis**

Haofeng Chen^#,1^, Haidong Yu^#,1^, Dongjiang Lin^#,1^, Guojun Yang^#,1^, Yuchen Xie^1^, Yang Zhang^1^, Jifeng Yuan*^,1^

^1^ State Key Laboratory of Cellular Stress Biology, School of Life Sciences, Faculty of Medicine and Life Sciences, Xiamen University, Fujian, China

^#^ These authors contributed equally to the experiments.

* Corresponding author Email: [jfyuan@xmu.edu.cn](mailto:jfyuan@xmu.edu.cn)

ORCRID: 0000-0003-1874-190X

**Supplementary Figures**


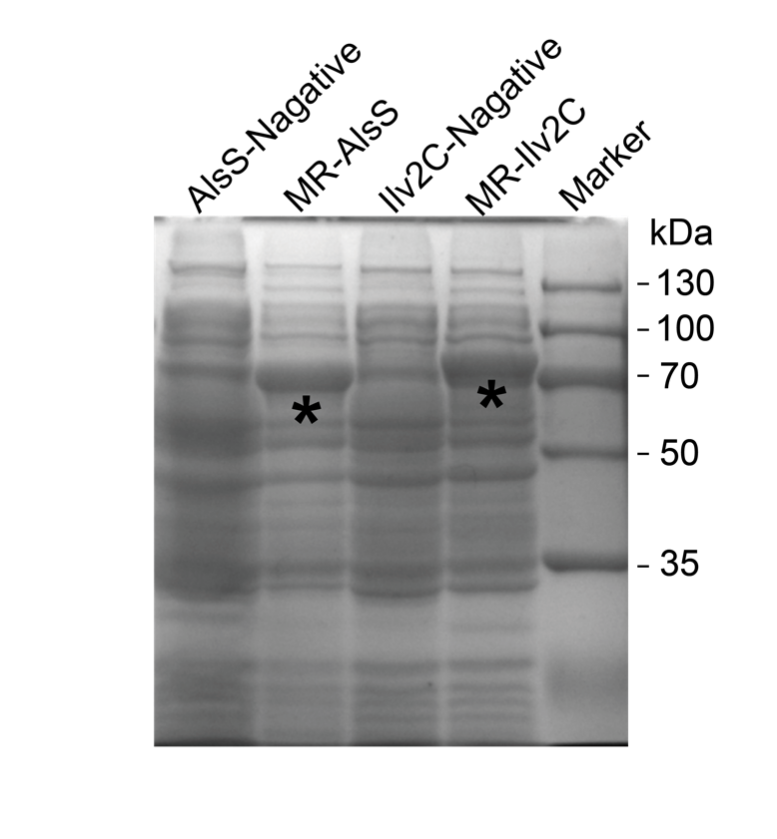


**Supplementary Figure S1.** Sodium dodecyl sulfate polyacrylamide gel electrophoresis (SDS-PAGE) image of whole-cell catalysts expressing AlsS and Ilv2C. AlsS (~62 kDa), Ilv2C (~74 kDa). The symbol of * indicates the successful expression of expected protein bands.


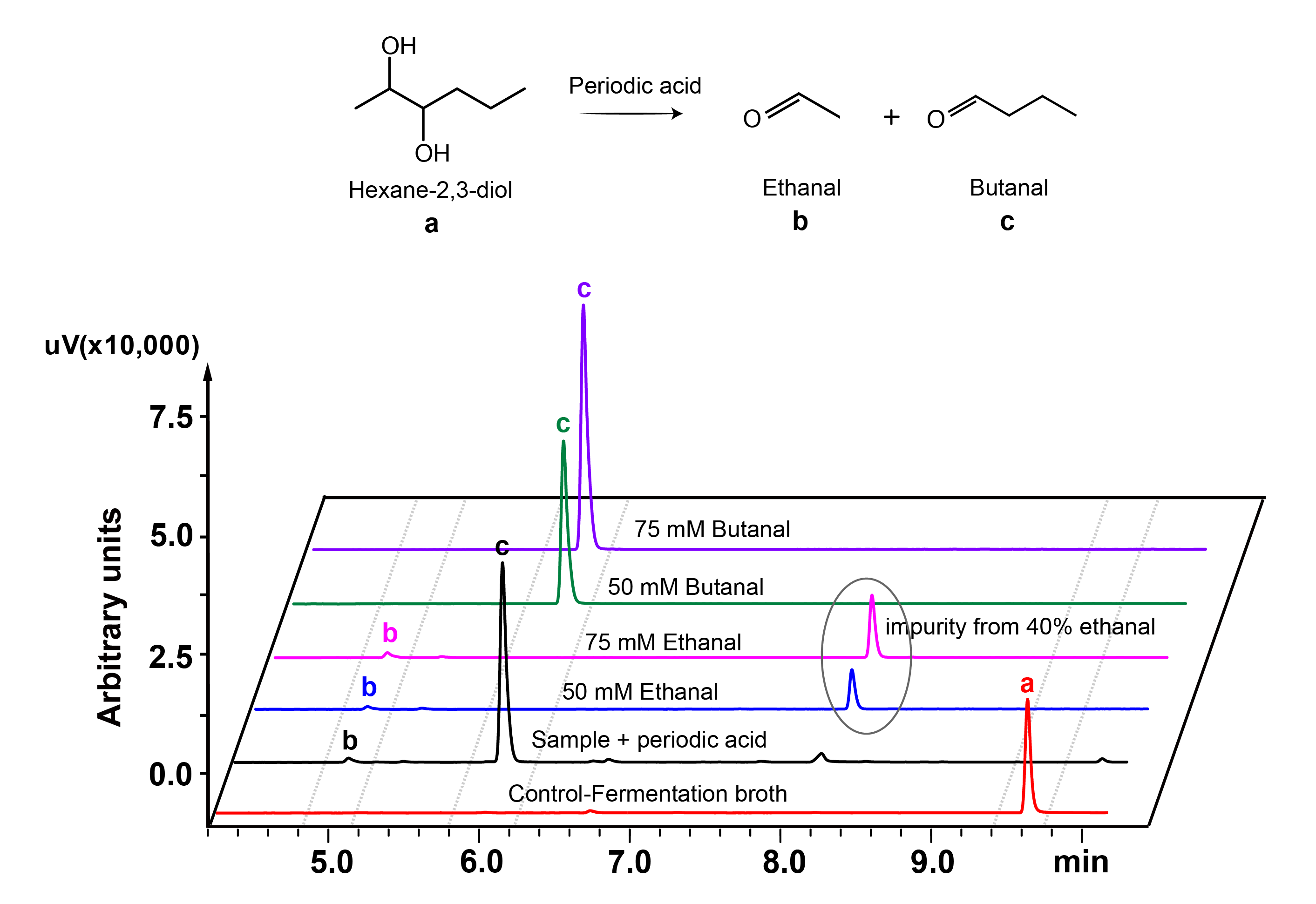


**Supplementary Figure S2.** Periodic acid treatment to confirm the identity of hexane-2,3-diol. The products obtained from the reaction between the sample and periodic acid were extracted with dodecane and subsequently analyzed by gas chromatography to verify whether the product was hexane-2,3-diol.

**
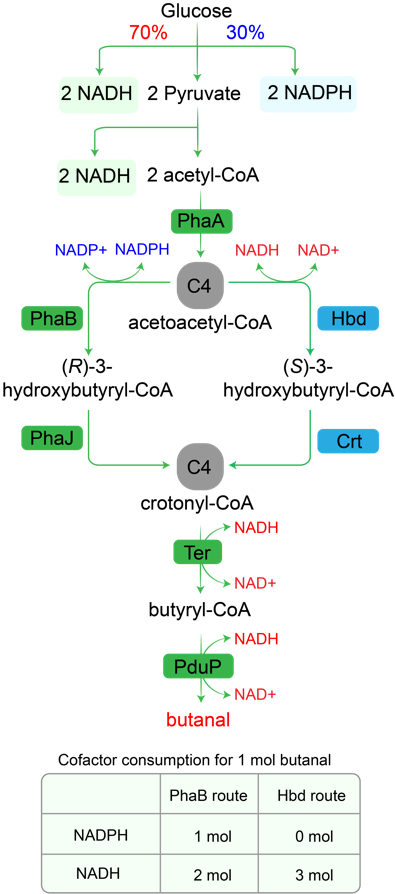
**

**Supplementary Figure S3.** Schematic diagram of the native *Clostridium* pathway and the redesigned *Clostridium*-derived clostridial pathway for butanal production. The central metabolism in *E. coli* typically generates NADPH/NADH at ~ 0.23. 1 mol of glucose is assumed to enter the pentose phosphate pathway at 30% and the glycolytic pathway at 70%, ultimately producing 2.6 mol of NADH and 0.6 mol of NADPH.

**
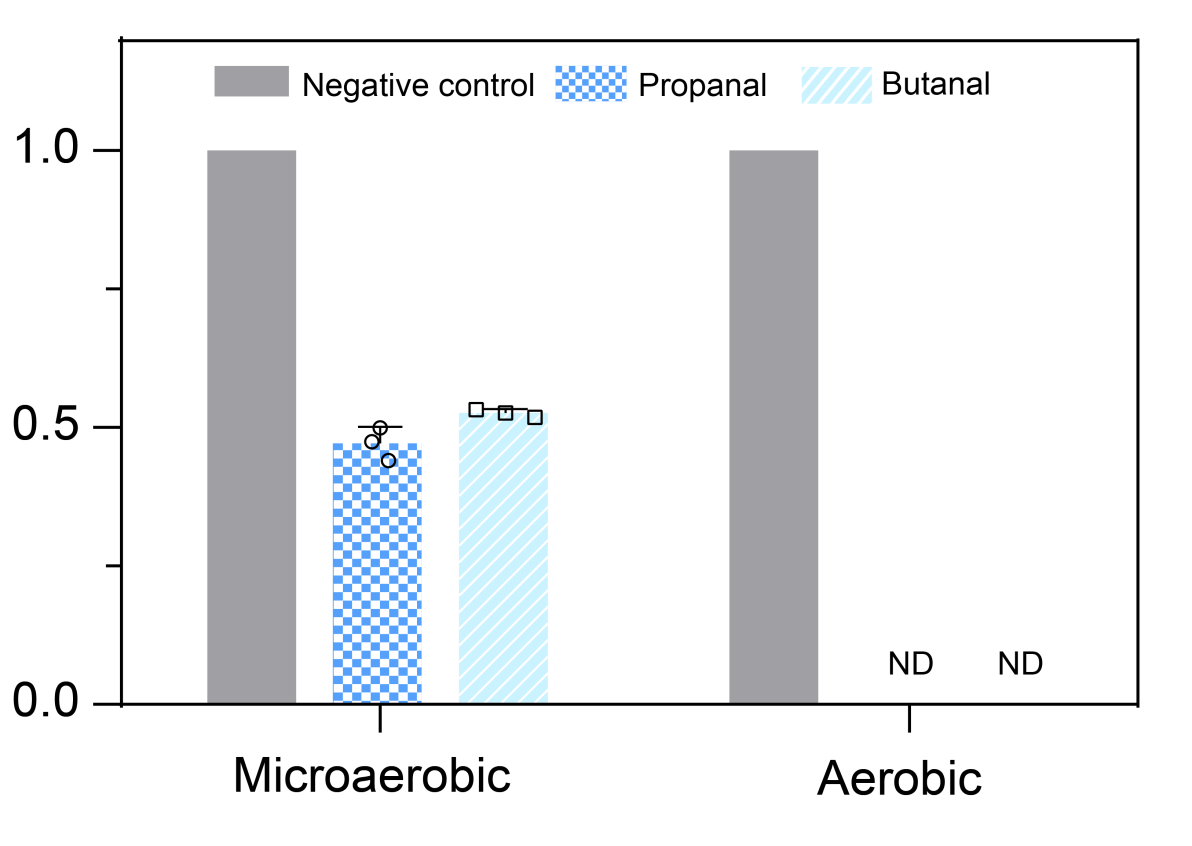
**

**Supplementary Figure S4.** Microaerobic and aerobic retainment of propanal and butanal. Propanal and butanal were supplied exogenously, and their accumulation at 0 h and 24 h was quantified under aerobic and microaerobic conditions. Negative control is the result of 0 h detection. The average and standard deviation are obtained from three biological replicates.

**
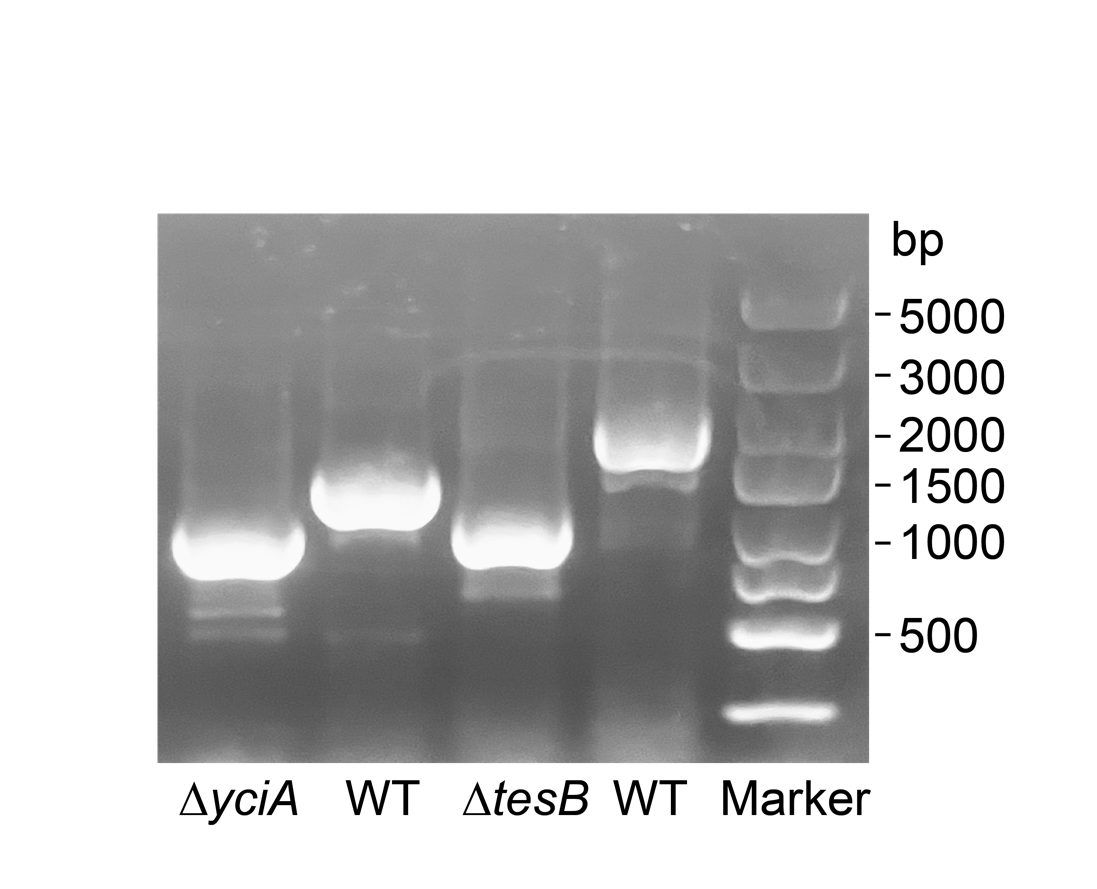
**

**Supplementary Figure S5.** Gel electrophoresis image of *yciA* and *tesB* gene knockout in the MR4.0 strain.

**
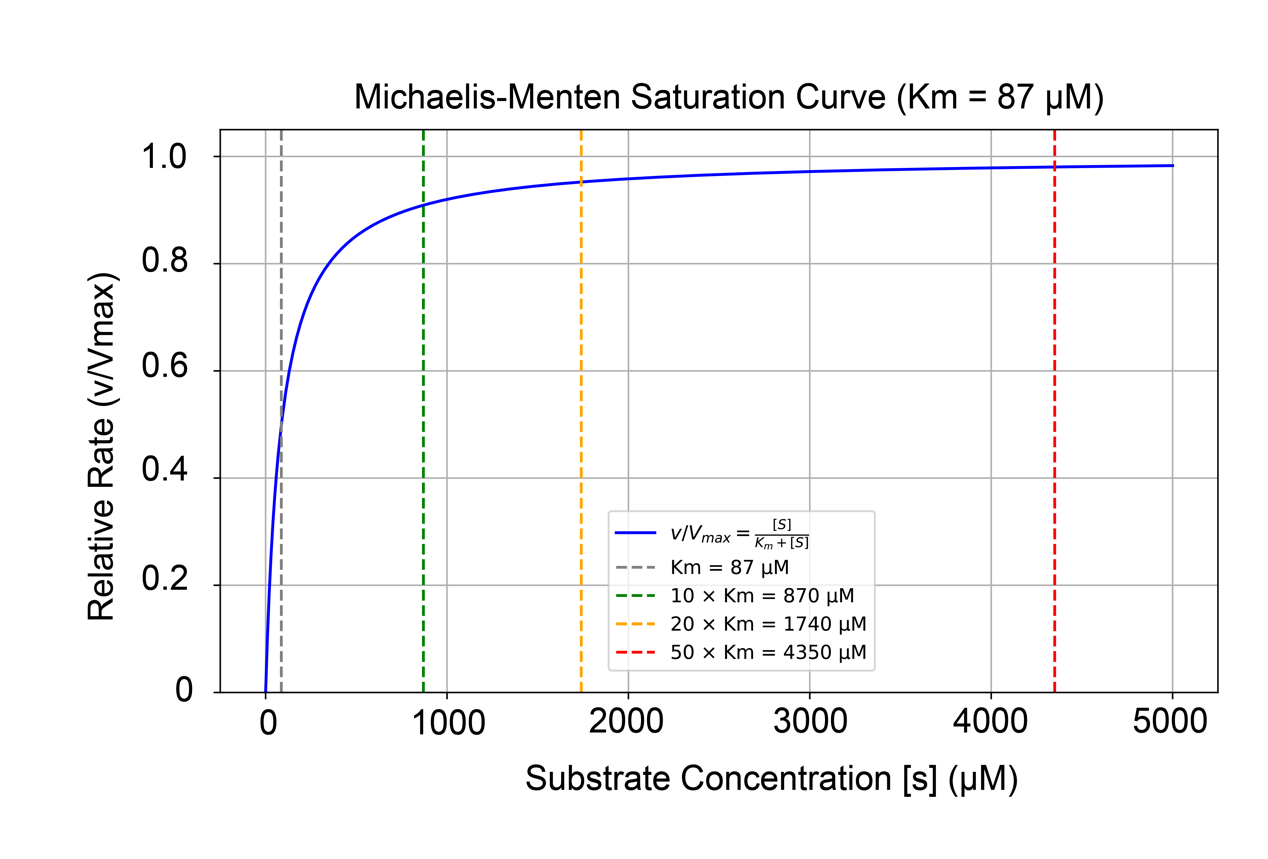
**

**Supplementary Figure S6.** The Michaelis-Menten equation to calculate the substrate concentration required for convergence to *V*max (*K*m=87 μM).

**
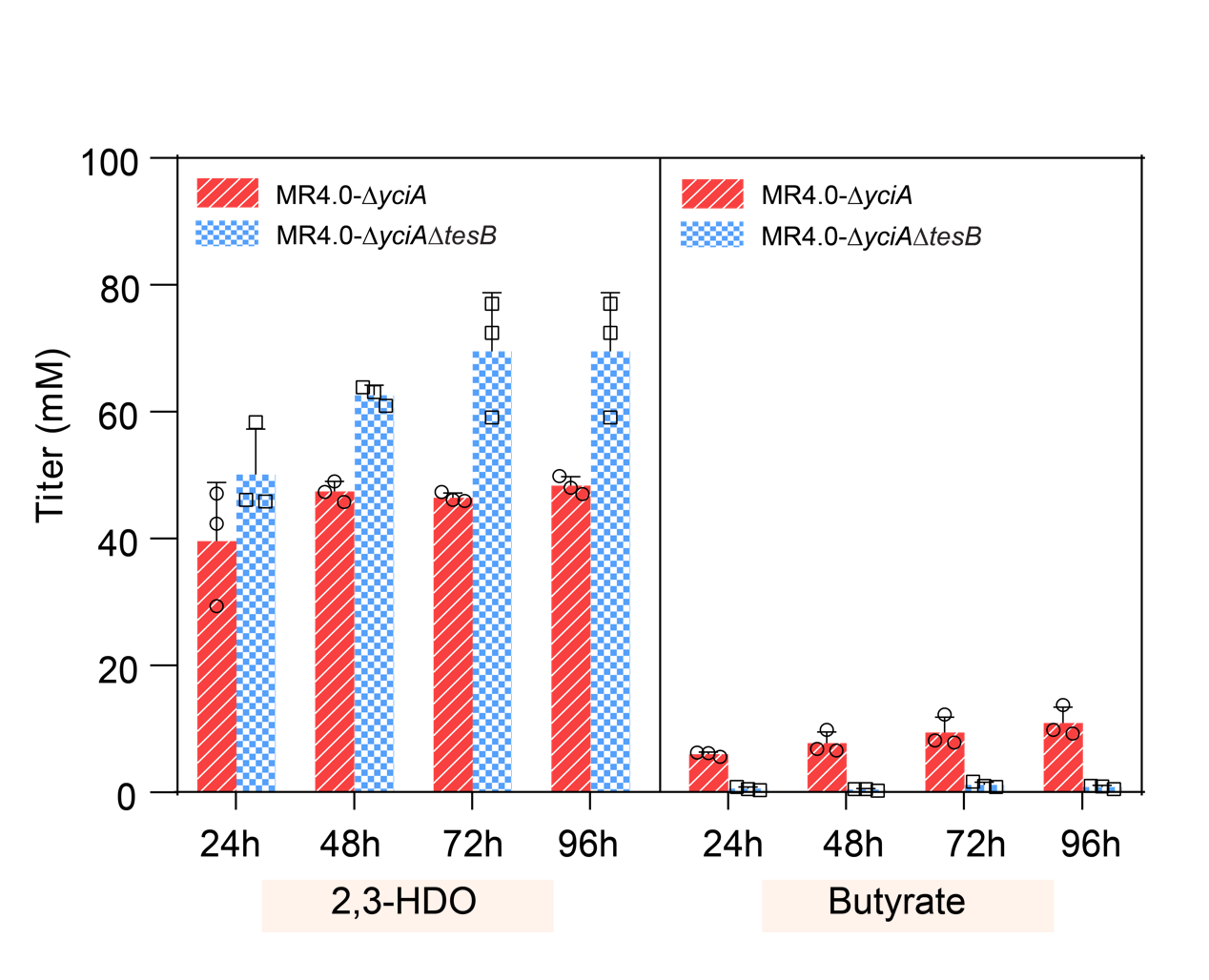
**

**Supplementary Figure S7.** The production of 2,3-HDO and butyrate by the PhaB-PhaJ route in the MR4.0*ΔyciA* and the MR4.0*ΔyciAΔtesB* strain. The average and standard deviation are obtained from three biological replicates.

**
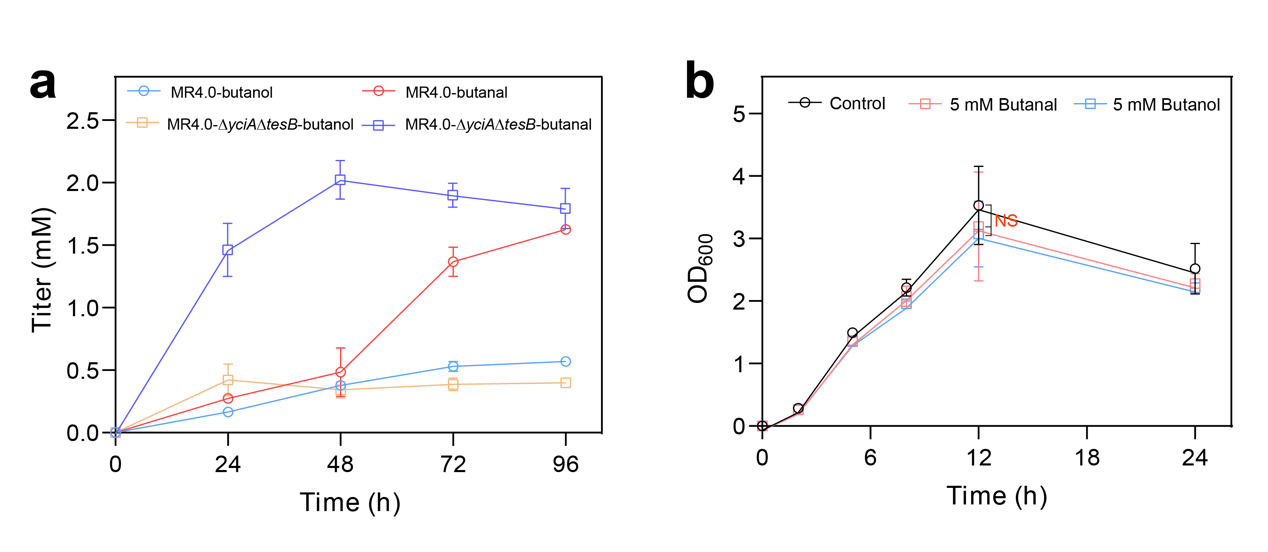
**

**Supplementary Figure S8.** Detection of butanol and butanal accumulation levels during fermentation of MR4.0 and MR4.0*ΔyciAΔtesB* (**a**). Fermentation conducted under microaerobic conditions in 20 ml M9 fermentation medium. Tolerance testing of MR4.0*ΔyciAΔtesB* strain towards butanal and butanol (**b**). Fermentation assay conducted in 48-well plates using 1 ml M9 fermentation medium. The average and standard deviation are obtained from three biological replicates.

**
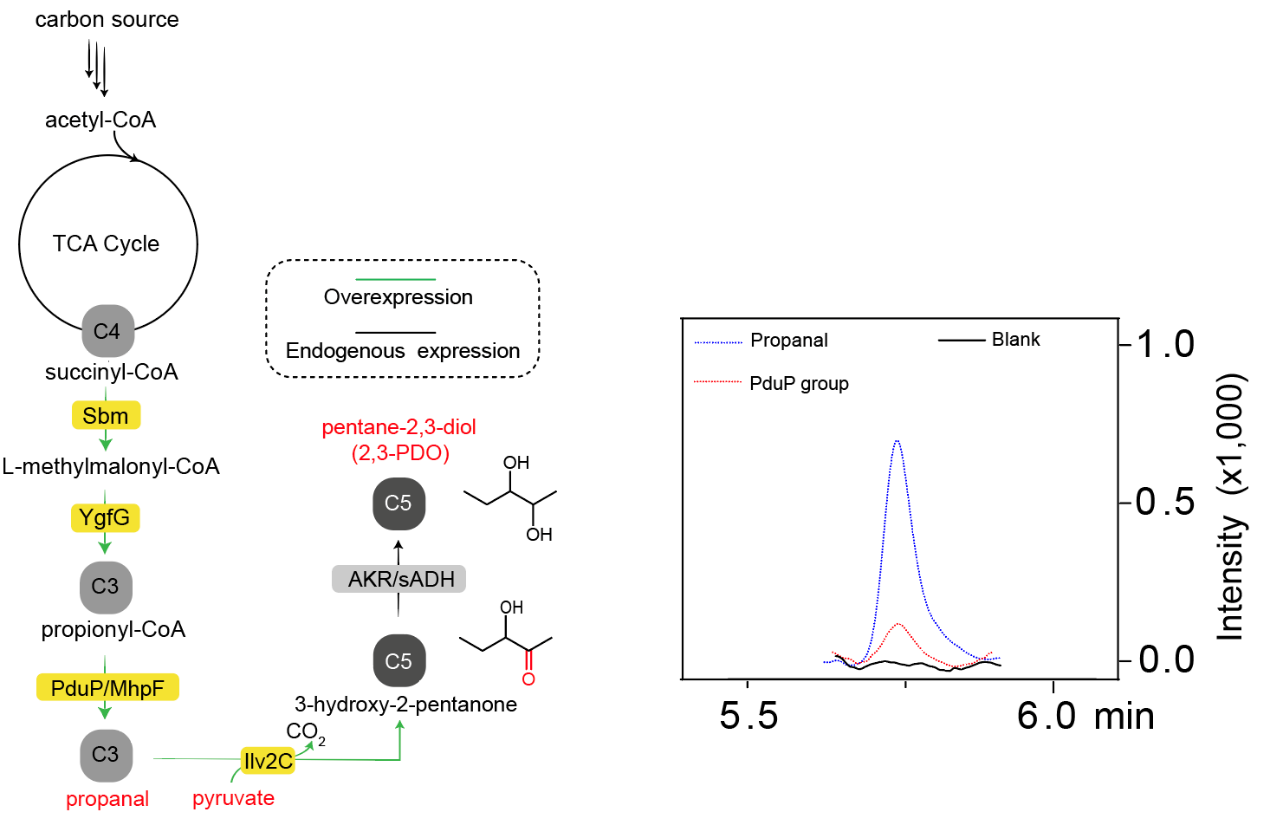
**

**Supplementary Figure S9.** The proposed pathway for the production of 2,3-PDO through the Sleeping Beauty mutase (Sbm) pathway and GC-FID analysis of propanal production.

**
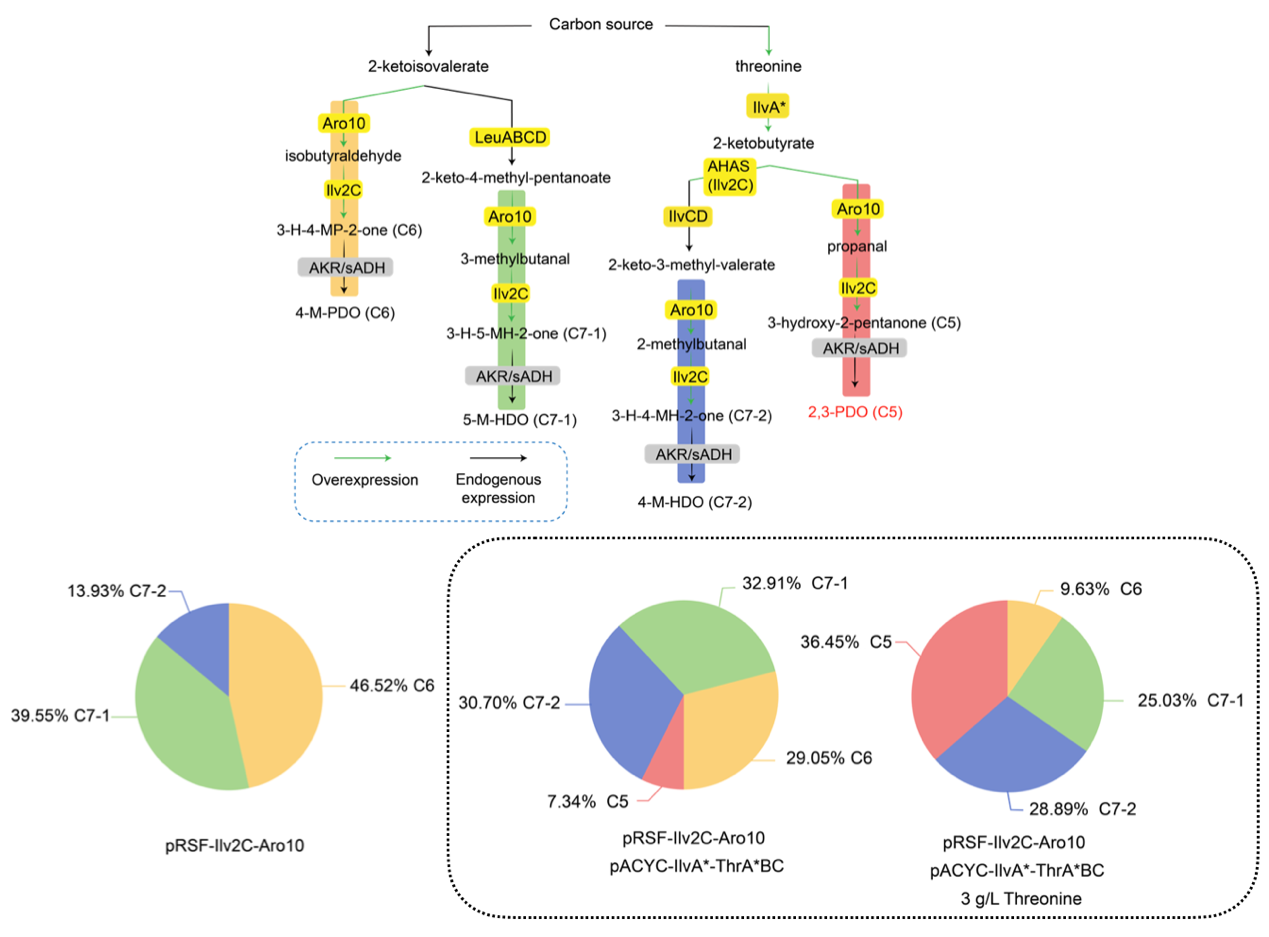
**

**Supplementary Figure S10.** The distribution profile of diols between different generations of strains. Overexpression of L-threonine metabolic pathway (IlvA*-ThrA*BC), and the external supplementation of L-threonine, achieved higher proportions of 2,3PDO.

**
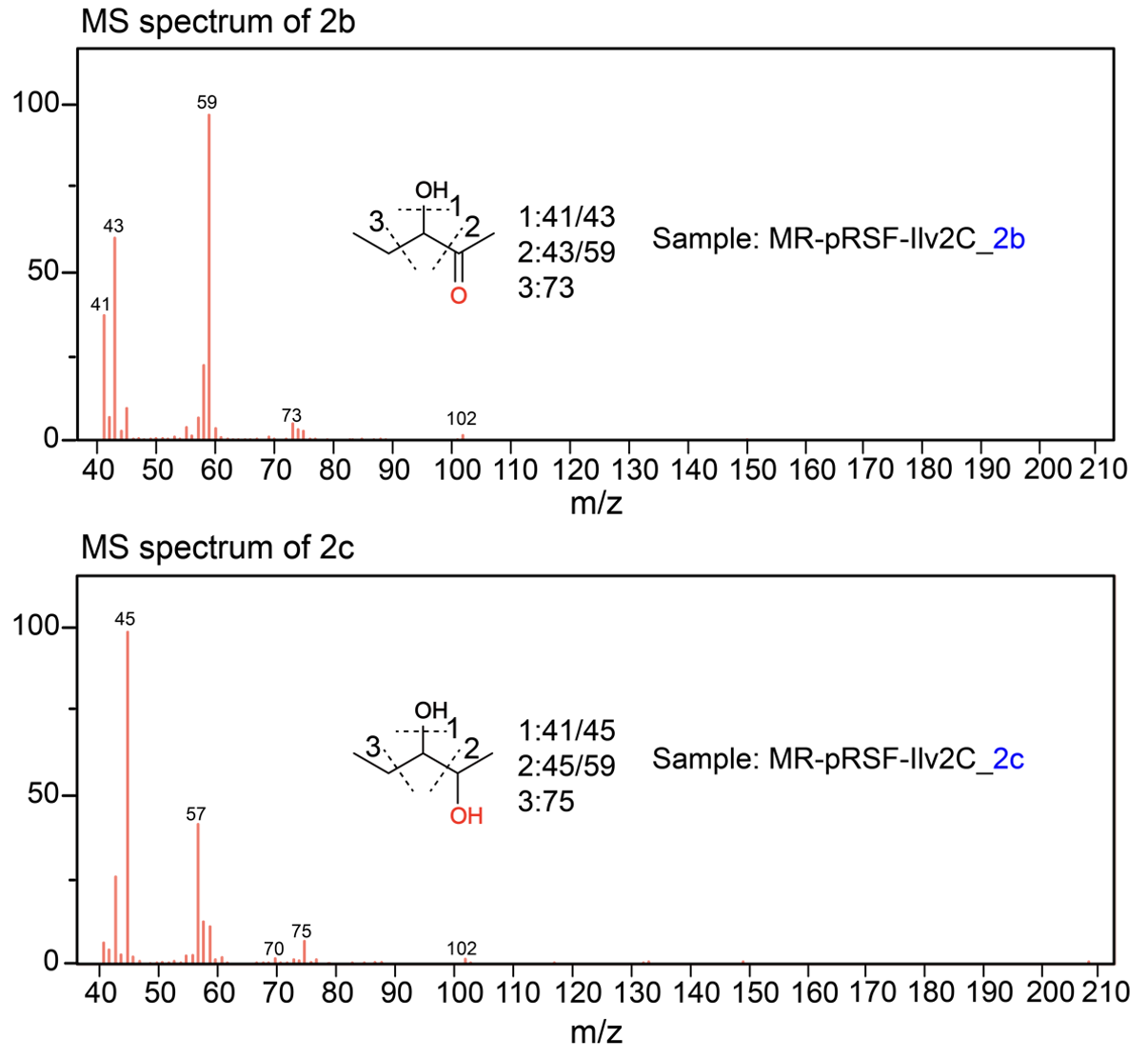
**

**Supplementary Figure S11.** Mass spectra of compound **2b** (3-hydroxypentan-2-one, 3-H-P-one) and compound **2c** (2,3-PDO) obtained by MR4.0-Ilv2C-Aro10-IlvA*-ThrA*BC.


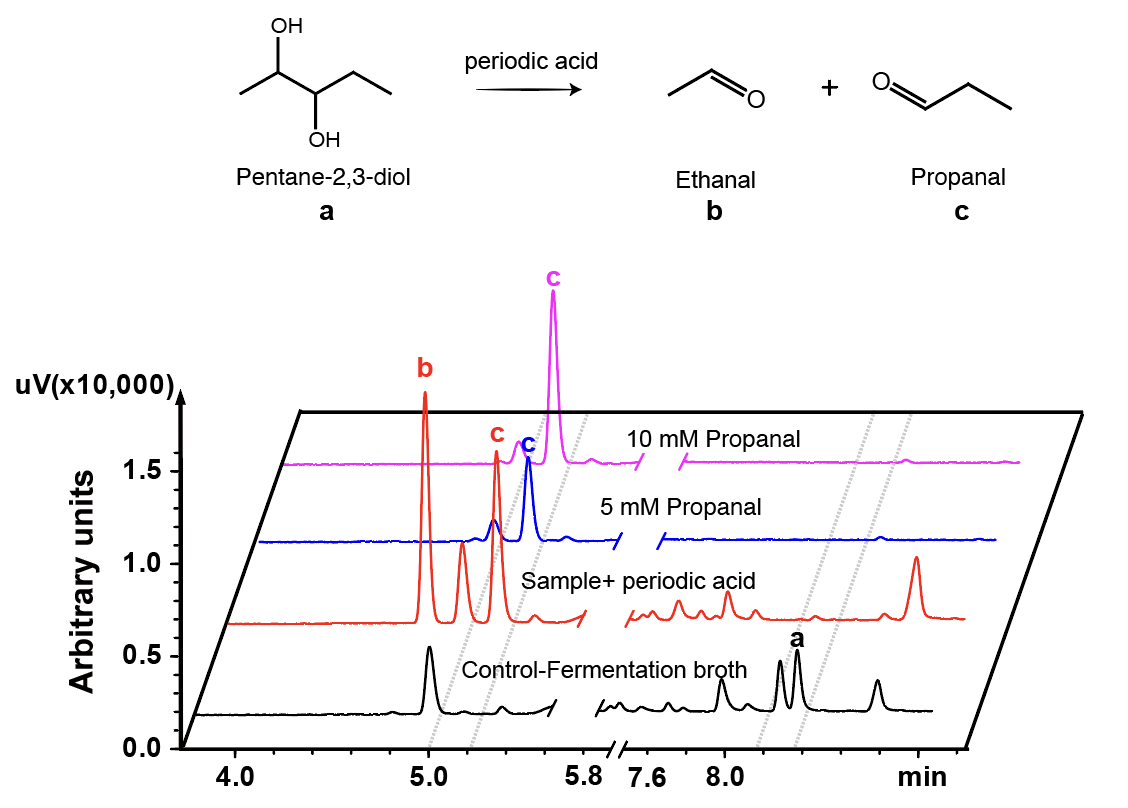


**Supplementary Figure S12.** Periodic acid treatment to confirm the identity of pentane-2,3-diol. The products obtained from the reaction between the sample and periodic acid were extracted with ethyl acetate and subsequently analyzed by gas chromatography to verify whether the product was pentane-2,3-diol.


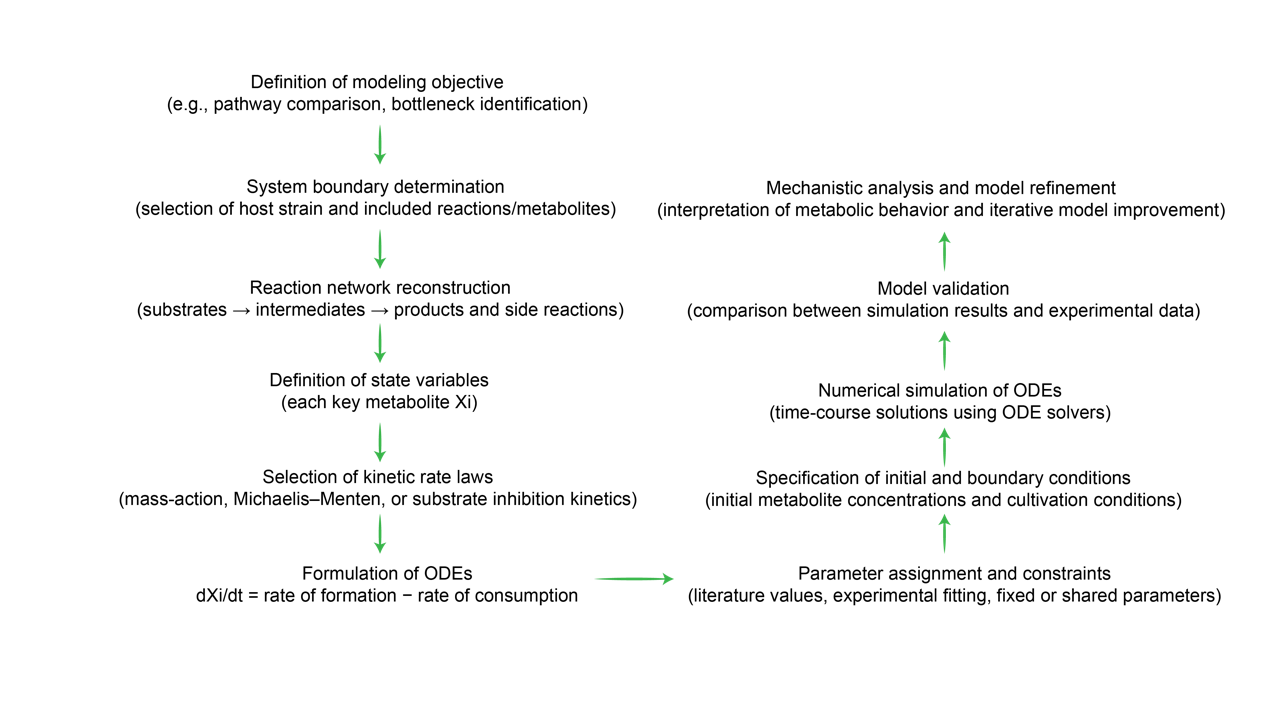
 **Supplementary Figure S13.** Ordinary Differential Equation (ODE) model construction process.

**Supplementary Table**

**Supplementary Table S1.** Fermentation yield data of representative diols.

| **Diols** | **Diol type** | **Representative microorganisms** | **Production method** | **Key performance parameters** | | | **Fermentation type** |
| --- | --- | --- | --- | --- | --- | --- | --- |
|  |  |  |  | Titer  (g/L) | Productivity (g/L/h) | Yield (mol/mol) |  |
| 1,3-propanediol (1,3-PDO) [1-2] | α,β-alkanediol | *Clostridium pasteurianum* G8 | Electrochemically mediated bio-conversion of glycerol | 120.67 | 4.83 | 0.59 | Electrochemically Assisted Anaerobic Fermentation + Downstream Integrated Separation  (Industrial fermentation level) |
|  |  | *E. coli* | Glycerol-dependent pathway using glycerol as substrate | 63.16 | 0.96 |  | Two-stage pH-controlled fed -batch fermentation  (2 L fermentation volume) |
| 1,4-butanediol (1,4-BDO) [3] | α,β-alkanediol | *E. coli* | Succinate-dependent pathway | 125 | 3.5 | 0.8 | Fed -batch fermentation  (Industrial fermentation level) |
| 1,3-butanediol (1,3-BDO) [4] | α,β-alkanediol | *E. coli* | Ac-CoA-dependent pathway | 71.1 |  | 0.65 | Fed -batch fermentation  (2.5 L fermentation volume) |
| 2,3-butanediol (2,3-BDO) [5] | α,β-alkanediol | *S. cerevisiae* | Replace ethanol biosynthetic pathway with a 2,3-BD-producing pathway | 180 | 2.64 |  | Fed-batch fermentation  (1L fermentation volume) |
| 4-methylpentane-2,3-diol [6] | branched-chain-β,γ-diol | *E. coli* | Amino acid metabolism | 15.3 | 0.106 | 0.486 | Shake flask fermentation  (500mL fermentation volume) |

| 2,3-Pentanediol (2,3-PDO) | β,γ-alkanediol | *E. coli* | Reversal β-oxidation pathways | 1.61 | 0.0168 | 0.070 | Shake flask fermentation  (500mL fermentation volume) |
| --- | --- | --- | --- | --- | --- | --- | --- |
| 2,3-Hexanediol (2,3-HDO) | β,γ-alkanediol | *E. coli* | Amino acid metabolism | 17.99 | 0.28 | 0.438 | Fed -batch fermentation (2L fermentation volume) |

**Supplementary Table S2.** List of oligonucleotides used in this study.

| Primer names | Sequences |
| --- | --- |
| IlvA_BamHI_fwd | CGCGGATCCGATGGCTGACTCGCAACCCC |
| IlvAfbr_OE_rev | GTGTTGGCGAAGCGCAGAAACGCGCCCGGTGATTCCGG |
| IlvAfbr_OE_fwd | GCGCGTTTCTGCGCTTCGCCAACACGCTGGGTACGTACTG |
| IlvA_BglX_rev | AGAGACTCGAGTCTCTCTTAGATCTCTCCTCTAACCCGCCAAAAAGAACC |
| ThrA*BC_BglII_fwd | AGCGCAGATCTGATGCGAGTGTTGAAGTTCGG |
| ThrA*BC_OE_rev | CAGCACCACGAAAATACGGGCGCGTGACATC |
| ThrA*BC_OE_fwd | GCCCGTATTTTCGTGGTGCTGATTACGCAATC |
| ThrA*BC_XhoI_rev | AGAGACTCGAGTTACTGATGATTCATCATCAATTTAC |
| Ilv2C_BsaI_fwd1 | TTGGTCTCGGATCCGATGCCAGAGCCTGCTCCAAG |
| Ilv2C_BsaI_rev1 | TTGGTCTCATCCTTCAGTGCTTACCGCCTGTAC |
| PduP_BsaI_fwd2 | TTGGTCTCAAGGAGATATATAATGAATACTTCTGAACTCGAAAC |
| PduP_BsaI_rev2 | TTGGTCTCCTCGAGTTAGCGAATAGAAAAGCCGTTGG |
| PhaA_BamHI_fwd | CGGGATCCAAAGGAGGACTACACAATGACTGACG |
| PhaB_XhoI_fwd | TGGTCTCCTCGAGTCTCCTGTAGATCTTTCCTCCTTTCAGCCCATATGCAGGCCGC |
| FadB_BsaI_fwd1 | TTGGTCTCGGATCCGATGCTTTACAAAGGCGACACC |
| FadB_BsaI_rev1 | TTGGTCTCCTCCTTTAAGCCGTTTTCAGGTCGCC |
| Ter-PhaA_BsaI_fwd2 | TTGGTCTCAAGGAGGAATTAAGCATGATAGTAAAACCCATG |
| Ter-PhaA_BsaI_rev2 | TTGGTCTCCTCGAGTCTCCTGTAGATCTTTCCTCCTTTTATTTGCGCTCGACTGCCAG |
| 1tesB_fwd | AGAGAGCGGCCGCCTTCAGTACGCACCGCTTTC |
| 2tesB_OE_rev | CTCGAGTCCTCTCTGGATCCGCGCCTGACTCATATAACTC |
| 3tesB_OE_fwd | GGATCCAGAGAGGACTCGAGAGCCGGAGGTGAAAACCGTC |
| 4tesB_rev | AGAGAGTCGACCACTGCTGGGGGCGTGTTCTG |
| 1yciA_fwd | AGAGAGCGGCCGCGATGGCCCTGATCACTTTTG |
| 2yciA_OE_rev | CTCGAGTCCTCTCTGGATCCGTTATGTGTTGTAGACATGG |
| 3yciA_OE_fwd | GGATCCAGAGAGGACTCGAGGTCAAAAGCCTCCGGTCGG |
| 4yciA_rev | AGAGAGTCGACTGGTCATTGAAAAGCCGAAG |
| Ec_gtesB-fwd | GCGGATCCTCGAGAAAACCGATGCCGTGGTTTTAGAGCTAGAAATAGC |
| Ec_gyciA-fwd | GCGGATCCTGTCCAGAAAGGGACGACATGTTTTAGAGCTAGAAATAGC |
| gRNA_rev | AGAGACTCGAGGATCAATACAAAAAAAGCACCGACTCGGTGCC |
| YdjG_BamHI_fwd | CGCGGATCCGatgaaaaagatacctttaggc |
| YdjG_XhoI_rev | CCGCTCGAGttaacgctccagggcctctgc |
| YdhF_BamHI_fwd | CGCGGATCCGatggttcagcgtattactattg |
| YdhF_XhoI_rev | CCGCTCGAGttacggtacgtcgtaccccag |
| YghZ_BamHI_fwd | CGCGGATCCGatggtctggttagcgaatcc |
| YghZ_XhoI_rev | CCGCTCGAGtcatttatcggaagacgcctg |

**Supplementary Table S3.** List of plasmids used in this study.

| Plasmids | Description | Source |
| --- | --- | --- |
| pRSFDuet1 | Expression vector | Novagen |
| pETDuet1 | Expression vector | Novagen |
| pACYCDuet1 | Expression vector | Novagen |
| pRSF-Ilv2C-Aro10 | pRSFDuet-1 derivative with insertion of *Ilv2C-Aro10* gene | This study |
| pRSF-Ilv2c-PduP | pRSFDuet-1 derivative with insertion of *Ilv2C-PduP* gene | This study |
| pET-YdjG | pETDuet-1 derivative with insertion of *YdjG* gene | This study |
| pET-YghZ | pETDuet-1 derivative with insertion of *YghZ* gene | This study |
| pET-YdhF | pETDuet-1 derivative with insertion of *YdhF* gene | This study |
| pET-TbsADH | pETDuet-1 derivative with insertion of *TbsADH* gene | This study |
| pET- CbsADH | pETDuet-1 derivative with insertion of *CbsADH* gene | This study |
| pET- LpsADH | pETDuet-1 derivative with insertion of *LpsADH* gene | This study |
| pET- PhaJ-Ter | pETDuet-1 derivative with insertion of *PhaJ-Ter* gene | This study |
| pET- PhaJ-Ter -PhaAB | pETDuet-1 derivative with insertion of *PhaJ-Ter-PhaAB* gene | This study |
| pET- FadB-Ter-PhaA | pETDuet-1 derivative with insertion of FadB*-Ter-PhaA* gene | This study |
| pACYC-IlvA*-ThrA*BC | pACYCDuet-1 derivative with insertion of *IlvA** and *ThrA*BC* genes | This study |
| pKD46-Cas9 | pKD46 derivative for Cas9, AmpR, pSC101 origin | Lab stock |
| pACYC-sacB | Vector for cloning gRNA, CamR, p15A origin | Lab stock |
| pACYC-sacB-gYciA | pAC-SacB derivative with gRNA-YciA | This study |
| pACYC-sacB-gTesB | pAC-SacB derivative with gRNA-TesB | This study |

**Supplementary Table S4.** List of strains used in this study.

| Stains | Abbreviation | Description | Source |
| --- | --- | --- | --- |
| *E. coli* TOP10 | TOP10 | For gene cloning | Lab stock |
| *E. coli* RARE | MR | For gene expression | Lab stock |
| *E. coli* MR4.0 | MR4.0 | RARE with *Pta*, *PflB*, *LdhA*, *AdhE* deletion | Lab stock |
| *E. coli* MR-Ilv2C | MR-Ilv2C | RARE strain harboring pRSF-Ilv2C | Lab stock |
| *E. coli* MR-AlsS | MR-AlsS | RARE strain harboring pRSF-AlsS | Lab stock |
| *E. coli* MR4.0-Δ*yciA* | MR4.0Δ*yciA* | RARE with *Pta*, *PflB*, *LdhA*, *AdhE, YciA* deletion | This study |
| *E. coli* MR4.0-Δ*yciA*Δ*tesB* | MR4.0Δ*yciA*Δ*tesB* | RARE with *Pta*, *PflB*, *LdhA*, *AdhE, YciA, TesB* deletion | This study |
| *E. coli* MR4.0-Ilv2c-PduP-PhaJ-Ter-PhaAB | MR4.0-Ilv2c-PduP-PhaJ-Ter-PhaAB | MR4.0 strain harboring pRSF-Ilv2c-PduP and pET-PhaJ-Ter-PhaAB | This study |
| *E. coli* MR4.0-Δ*yciA*-Ilv2c-PduP-PhaJ-Ter-PhaAB | MR4.0Δ*yciA*-Ilv2c-PduP-PhaJ-Ter-PhaAB | MR4.0Δ*yciA* strain harboring pRSF-Ilv2c-PduP and pET-PhaJ-Ter-PhaAB | This study |
| *E. coli* MR4.0-Δ*yciA*Δ*tesB*-Ilv2c-PduP-PhaJ-Ter-PhaAB | MR4.0Δ*yciA*Δ*tesB*-Ilv2c-PduP-PhaJ-Ter-PhaAB | MR4.0Δ*yciA*Δ*tesB* strain harboring pRSF-Ilv2c-PduP and pET-PhaJ-Ter-PhaAB | This study |
| *E. coli* MR4.0-Ilv2c-PduP-FadB-Ter-PhaA | MR4.0-Ilv2c-PduP-FadB-Ter-PhaA | MR4.0 strain harboring pRSF-Ilv2c-PduP and pET-FadB-Ter-PhaA | This study |
| *E. coli* MR4.0-Δ*yciA*Δ*tesB*-Ilv2c-PduP-FadB-Ter-PhaA | MR4.0Δ*yciA*Δ*tesB*-Ilv2c-PduP-FadB-Ter-PhaA | MR4.0Δ*yciA*Δ*tesB* strain harboring pRSF-Ilv2c-PduP and pET-FadB-Ter-PhaA | This study |
| *E. coli* MR4.0-Ilv2c-Aro10-IlvA*-ThrA*BC | MR4.0-Ilv2c-Aro10-IlvA*-ThrA*BC | MR4.0 strain harboring pRSF-Ilv2c-Aro10 and pACYC-IlvA*-ThrA*BC | This study |
| *E. coli* MR4.0-Ilv2c-Aro10-IlvA*-ThrA*BC-YdjG | MR4.0-Ilv2c-Aro10-IlvA*-ThrA*BC-YdjG | MR4.0 strain harboring pRSF-Ilv2c-Aro10, pACYC-IlvA*-ThrA*BC and pET-YdjG | This study |
| *E. coli MR4.0-Ilv2c-Aro10-IlvA*-ThrA*BC-*YghZ | MR4.0-Ilv2c-Aro10-IlvA*-ThrA*BC-YghZ | MR4.0 strain harboring pRSF-Ilv2c-Aro10, pACYC-IlvA*-ThrA*BC and pET-YghZ | This study |
| *E. coli* MR4.0-Ilv2c-Aro10-IlvA*-ThrA*BC-YdhF | MR4.0-Ilv2c-Aro10-IlvA*-ThrA*BC-YdhF | MR4.0 strain harboring pRSF-Ilv2c-Aro10, pACYC-IlvA*-ThrA*BC and pET-YdhF | This study |
| *E. coli* MR4.0-Ilv2c-Aro10-IlvA*-ThrA*BC-TbsADH | MR4.0-Ilv2c-Aro10-IlvA*-ThrA*BC-TbsADH | MR4.0 strain harboring pRSF-Ilv2c-Aro10, pACYC-IlvA*-ThrA*BC and pET-TbsADH | This study |
| *E. coli* MR4.0-Ilv2c-Aro10-IlvA*-ThrA*BC-CbsADH | MR4.0-Ilv2c-Aro10-IlvA*-ThrA*BC-CbsADH | MR4.0 strain harboring pRSF-Ilv2c-Aro10, pACYC-IlvA*-ThrA*BC and pET-CbsADH | This study |
| *E. coli* MR4.0-Ilv2c-Aro10-IlvA*-ThrA*BC-LpsADH | MR4.0-Ilv2c-Aro10-IlvA*-ThrA*BC-LpsADH | MR4.0 strain harboring pRSF-Ilv2c-Aro10, pACYC-IlvA*-ThrA*BC and pET-LpsADH | This study |

**Supplementary Table S5.** The values of the reaction constants for ODE simulation.

| Parameters | Definition | Units | Value |
| --- | --- | --- | --- |
| Ka1 | The formation rate of butyryl-CoA of PhaB-PhaJ route | 1/h | 0.011027 |
| Km1 | MR4.0: butanal formation rate of PhaB-PhaJ route | 1/h | 0.005785 |
| Ka2 | MR4.0: butyrate formation rate of PhaB-PhaJ route | 1/h | 0.004671 |
| Ka3 | Formation rate of hexane-2,3-diol | 1/h | 0.735194 |
| Km2 | MR4.0△*tesB*△*yciA*: butanal formation rate of PhaB- PhaJ route | 1/h | 0.024877 |
| Vmax | Maximum reaction rate | mmol/L/h | 3.14035358 |
| Ki | Inhibition constant | mmol/L | 2.59587953 |
| Kb1 | The formation rate of butyryl-CoA of FadB route | 1/h | 0.005 |

**Supplementary references**

[1] C. Zhang, P. Traitrongsat, A.-P. Zeng, *Bioprocess and Biosystems Engineering* **2023**, *46* (4), 565, <https://doi.org/10.1007/s00449-022-02841-6>.

[2] Y. Zhang, J. Yun, H. M. Zabed, Y. Dou, G. Zhang, M. Zhao, M. J. Taherzadeh, A. Ragauskas, X. Qi, *Bioresource Technology* **2023**, *369*, <https://doi.org/10.1016/j.biortech.2022.128438>.

[3] A. Burgard, M. J. Burk, R. Osterhout, S. Van Dien, H. Yim, *Current Opinion in Biotechnology* **2016**, *42*, 118, <https://doi.org/10.1016/j.copbio.2016.04.016>.

[4] T. Islam, T. P. Nguyen-Vo, V. K. Gaur, J. Lee, S. Park, *Bioresource Technology* **2023**, *376*, <https://doi.org/10.1016/j.biortech.2023.128911>.

[5] Y.-G. Lee, J.-H. Seo, *Biotechnology for Biofuels* **2019**, *12* (1), <https://doi.org/10.1186/s13068-019-1545-1>.

[6] P. Wu, H. Chen, Y. Chen, Y. Zhang, J. Yuan, *Nature Communications* **2025**, *16* (1), <https://doi.org/10.1038/s41467-025-59753-8>.
